# Application and challenges of tumor organoid technology in precision immunotherapy

**DOI:** 10.3389/fimmu.2025.1723995

**Published:** 2025-12-19

**Authors:** Xiaoming Zhao, Jun Gao, Yinan Liu, Yawen Shi, Meng Zhang, Chen Zhang, Jinghong Chen

**Affiliations:** 1 Department of Orthopaedics of the First Affiliated Hospital, Xi’an Jiaotong University, Xi’an, Shaanxi, China; 2 Key Laboratory of Environment and Genes Related to Diseases in the Education Ministry and National Health Commission (NHC) Key Laboratory of Environment and Endemic Diseases, School of Public Health, Xi’an Jiaotong University, Xi’an, Shaanxi, China

**Keywords:** immune microenvironment, model standardization, personalized therapy, precision immunotherapy, tumor organoids

## Abstract

In recent years, tumor organoid technology has emerged as a crucial bridge connecting basic research and clinical applications with a deeper understanding of tumor biology. This technology enables reconstruction of tumor-native structures *in vitro* and their microenvironments, providing new possibilities for assessing individual responses and optimizing treatment strategies. This paper details the application prospects of organoid technology in tumor immune microenvironment reconstruction, personalized therapy, and drug screening. It also analyzes the current challenges faced by organoid technology in clinical translation, including model standardization, the integrity of immune microenvironment reconstruction, and the importance of interdisciplinary collaboration. Furthermore, we discuss how emerging technologies, such as 3D bioprinting and microfluidic chips, are driving advancements in tumor research. In the future, tumor organoid technology will used to support precision immunotherapy by establishing standardized processes and databases to enhance data comparability and reproducibility. creating a closed-loop system of “patient-organoid-multi-omics data-clinical decision support” will further promote the development of precision medicine and facilitate clinical application.

## 1 Introduction

Over the past few decades, traditional two-dimensional (2D) cell culture methods have served as the primary platform for tumor biology research and drug screening due to their ease of use, low cost, and scalability (1–3). Researchers can rapidly cultivate large populations of homogenous cells on the surface of plastic culture dishes or flasks, allowing for in-depth exploration of fundamental characteristics such as tumor cell proliferation, migration, invasion, and drug sensitivity using a variety of established molecular and cellular techniques (4, 5). However, the inherent structural limitations of 2D adherent cultures have become increasingly apparent. Firstly, these systems cannot reconstruct the spatial polarity and gradient molecular distribution of cells within the three-dimensional (3D) architecture of *in vivo* tissues, leading to significant deviations in cell morphology, adhesion, and signal transduction pathways (6, 7). Secondly, the absence of authentic cell–cell and cell–matrix interactions makes it challenging to replicate the complex biological processes that involve multiple cell types and extracellular matrix (ECM) components in real tissue structures (8). Furthermore, as a typical culture system for a single tumor cell subtype, the 2D model fails to simulate the multifaceted interactions of immune cells, stromal cells, vascular networks, and inflammatory factors present in the tumor microenvironment (TME) (9). Consequently, studies on drug sensitivity and antitumor mechanisms derived from this model often struggle to make accurate predictions when translated to clinical settings (10).

To overcome the limitations of two-dimensional cell culture, animal models, particularly patient-derived xenograft (PDX) models, have been widely utilized in tumor biology and new drug development. PDX models maintain the complex interactions between transplanted tissue and host within an *in vivo* three-dimensional structure by implanting patient tumor tissues either orthotopically or ectopically into immunodeficient mice. This approach effectively preserves the genomic and phenotypic characteristics of the primary tumor, facilitating processes such as angiogenesis, stromal support, and partial recapitulation of *in situ* signaling pathways (11–13).However, PDX models also exhibit notable shortcomings. Their reliance on immunodeficient or humanized mice limits the assessment of bidirectional interactions between human immune cells and tumor cells. The success rate of transplantation is heavily influenced by the quality of donor tissue and the genetic background of the mouse strains, with construction cycles extending from several months to half a year, resulting in high costs and ethical concerns (14). Additionally, due to interspecies differences in immunity and metabolism, PDX models show limited efficacy in studying the mechanisms of immunotherapy and evaluating immunogenicity. In summary, while traditional 2D cell culture and PDX models each have their advantages, they also possess distinct limitations (15, 16). In particular, there is an urgent need for new technological breakthroughs to fill the gap in existing research platforms, especially in the context of precision immunotherapy, which demands high fidelity, low-cost, high-throughput, and reproducible *in vitro* models that can accurately replicate the immune microenvironment.

To establish a more *in vivo*-like three-dimensional tumor microenvironment *in vitro*, the scientific community has gradually developed 3D cell culture techniques. Early 3D cultures primarily relied on low-attachment culture plates or scaffold materials, allowing cells to spontaneously aggregate into spheroids under the influence of gravity; however, these methods often had limited efficacy in maintaining long-term functionality and expression (17, 18). In 2009, Sato et al. first reported the successful *in vitro* culture of mouse and human intestinal stem cells, constructing intestinal organoids within Matrigel that possessed self-renewal and multipotent differentiation capabilities. This achievement marked a milestone in organoid research (19). Subsequently, researchers expanded this technology to various organs, including breast, liver, pancreas, and lung tissues, gradually establishing a repository of organoids representing multiple tumor types (20–22).

**Figure 1:**
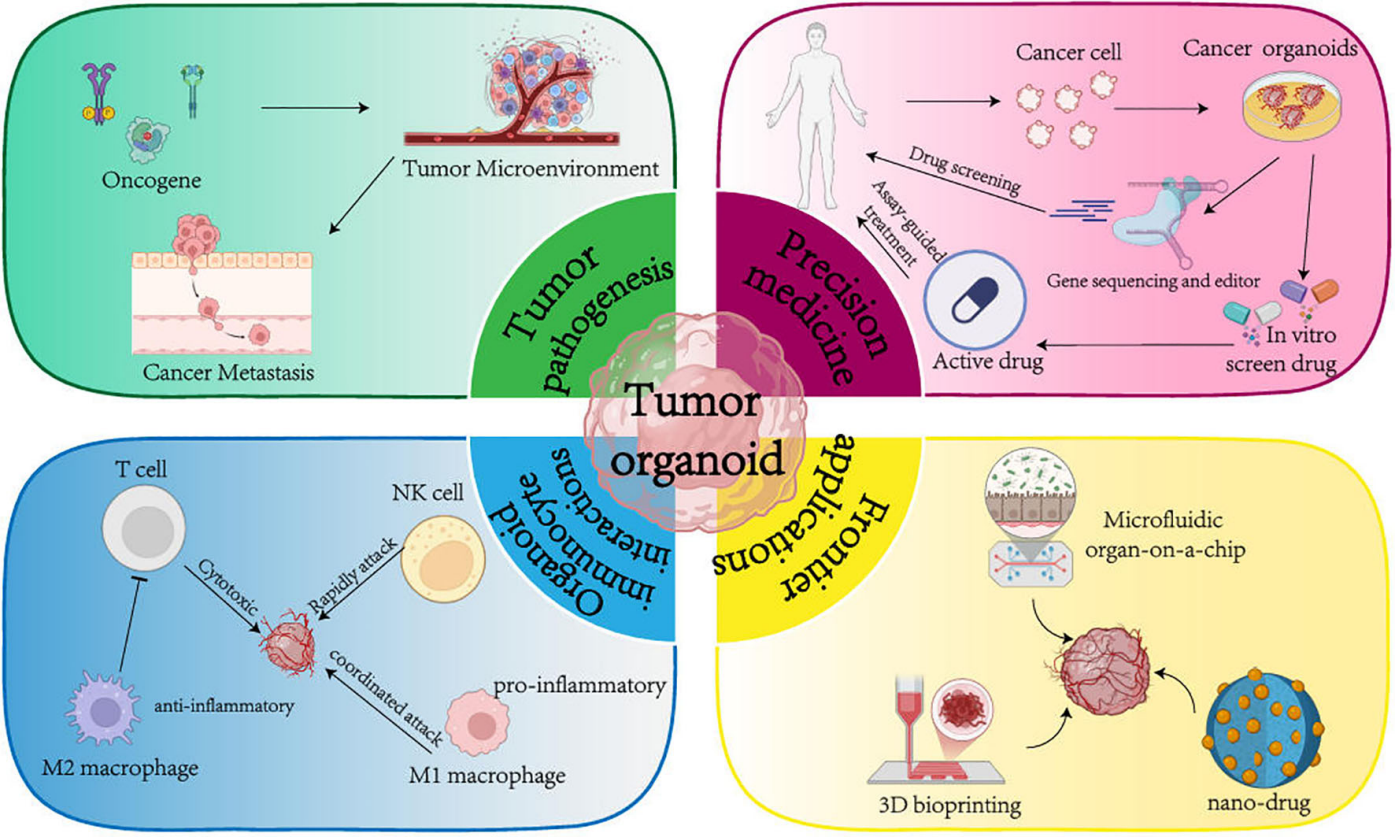
Exploration and application of tumor organoid technology in precision immunotherapy.

The core advantages of tumor organoids over traditional models are reflected in several key aspects: First, maintenance of three-dimensional structure. Organoids grow within Matrigel, reconstructing the multilayered spatial architecture of tumors, allowing cells to exhibit more native polarity and morphology in a three-dimensional environment (23). Second, preservation of heterogeneity. Tumor tissues derived from primary or metastatic sites inherently include various tumor cell subpopulations and corresponding driver gene mutation profiles, enabling the coexistence of clonal evolution and resistant subpopulations *in vitro* (24). Third, reconstruction of the local microenvironment. Researchers can introduce immune cells, stromal fibroblasts, vascular endothelial cells, and other tumor-associated components into the organoid culture system, creating “immune-enhanced” or “stroma-rich” organoid models to better simulate the complex cell–cell and cell–matrix interactions found *in vivo* (25). Fourth, high-throughput compatibility. The typical culture period for organoids ranges from 2 to 4 weeks, and the culture conditions can be adapted to microplate formats, making them compatible with automated liquid handling systems and high-throughput sampling and analysis. This provides an efficient platform for personalized drug sensitivity testing and large-scale drug screening (26). Therefore, organoid technology has transcended the limitations of traditional models in terms of personalization and predictability by virtue of its ability to faithfully recapitulate patient-specific tumor heterogeneity, the complex tumor microenvironment, and dynamic immune interactions *in vitro*. This offers a unique and unprecedented platform for advancing truly personalized precision immunotherapy for individual patients, encompassing efficacy prediction, investigation of resistance mechanisms, and screening of combination treatment regimens (See **Figure 1**).

## 2 Comparative analysis of tumor organoid technologies and challenges in precision immunotherapy

### 2.1 Comparative analysis of model systems: advantages and disadvantages of organoids versus traditional techniques

To comprehensively evaluate the applicability of different models in tumor research and drug screening, several key dimensions can be compared: culture duration, cost investment, success rates, preservation of heterogeneity, depth of immune microenvironment reconstruction, and high-throughput compatibility. Immortalized 2D cell lines have the most advantages in terms of culture duration, typically yielding large quantities of cells within a few days to a week, at the lowest cost and highest throughput. They are suitable for large-scale screening and gene function validation; however, their fidelity is the lowest, making it difficult to reflect the polyclonal characteristics and complex microenvironment of *in vivo* tumors (27–30).

In contrast, PDX models excel at preserving the original tissue structure and microenvironment of patient tumors, but they come with long construction cycles (ranging from several months to half a year) and high costs. Additionally, the requirement for immunodeficient mice limits their utility in studying immune mechanisms and evaluating immunotherapy (31, 32). Tumor organoid models fall between these two extremes, with culture periods of approximately 2 to 4 weeks, moderate costs, and high success rates. They can simulate three-dimensional structures and some aspects of the immune microenvironment *in vitro*, offering both high throughput and strong fidelity (33, 34). This makes them an ideal platform for predicting personalized treatment responses and conducting mid-scale drug screening.

### 2.2 Existing technological bottlenecks: challenges in microenvironment reconstruction and standardization

Despite the advantages of tumor organoids over traditional models, they still face significant technological bottlenecks in reconstructing the integrity of TME (27, 35–37). Firstly, the reconstruction of vascular networks and the physical-mechanical environment is still underdeveloped. Current mainstream matrix gel systems struggle to simulate *in vivo* hemodynamics and oxygen gradients, which impacts drug penetration studies and the exploration of hypoxia-related mechanisms (28, 38). Secondly, in immune co-culture systems, maintaining the long-term survival and functional activity of immune cells (such as effector T cells, macrophages, and natural killer cells) within a three-dimensional matrix, while ensuring authentic dynamic interactions with tumor cells, remains a significant challenge (31, 39). Moreover, there are notable differences among laboratories in terms of culture medium components, matrix formulations, cell seeding densities, and culture conditions, leading to a lack of unified standard operating procedures (SOPs) and quality control standards. This results in inadequate reproducibility of models and comparability of data. Specifically, the current challenge in standardizing organoid models lies in a fundamental systemic contradiction: the inherent nature of tumor heterogeneity necessitates personalized culture protocols, as different cancer types—or even subtypes within the same cancer—may require significantly distinct culture conditions. This inherent need for customization makes it extremely difficult to establish universal “one-size-fits-all” standards, creating a core conflict with the very objective of standardization, which strives for general applicability (33). At the technical level, heavy reliance on key reagents with complex compositions and significant batch-to-batch variations—such as Matrigel—introduces substantial variability, undermining experimental reproducibility. More critically, existing quality control standards predominantly focus on “static” parameters like morphology and genotype, while lacking a unified system for evaluating “functional fidelity,” such as drug response and immune interactions (28, 31).Consequently, most standard operating procedures developed by individual laboratories have not been validated through large-scale, multi-center studies, casting doubt on their general applicability and clinical relevance. Additionally, there is currently no established multidimensional assessment framework covering morphology, genomic stability, and functional testing, which limits the widespread application of organoid models in cross-center collaborative research and preclinical validation (33, 40). To address these shortcomings, efforts are urgently needed to optimize matrices, improve co-culture conditions, and establish standardized platforms, providing robust support for the in-depth application of tumor organoids in precision immunotherapy.

In summary, while traditional 2D cell cultures and PDX models each have their strengths, they struggle to balance fidelity and throughput. Tumor organoid technology offers a new opportunity for precision immunotherapy research, thanks to its comprehensive advantages in maintaining three-dimensional structure, preserving heterogeneity, and partially reconstructing the immune microenvironment. A comparative classification of different models is presented in **Table 1**. However, to achieve an efficient feedback loop from *in vitro* models to clinical decision-making, it is essential to continue addressing key issues related to TME integrity, immune co-culture, and standardization processes, laying a solid foundation for the in-depth discussion of model construction and optimization strategies in subsequent chapters.

**Table 1:** Comparative analysis of tumor organoid models and traditional models.

Comparison dimension	2D cell line	PDX model	Tumor organoids	References
Culture Period	3–7 days	4–8 months	2–4 weeks	(41–43)
Cost	Low	High	Moderate	(12, 44, 45)
Construction Success Rate	>95%	30-60%	70-85%	(43, 45–48)
Heterogeneity Retention	Poor (Monoclonal)	Excellent (Retains primary features)	Good (Polyclonal subpopulations)	(33, 49, 50)
Immune Microenvironment Reconstruction	Not achievable	Limited (Lacks human immunity)	Partially achievable (Requires co-culture)	(28, 31, 33, 50)
High-Throughput Compatibility	Excellent (96/384-well plates)	Poor (Limited by animal experiments)	Good (24/96-well plates)	(43, 51, 52)
Applicable Scenarios	Primary screening/Mechanistic studies	Preclinical validation	Personalized drug sensitivity/Immunotherapy research	(49, 53–55)

### 2.3 Challenges of tumor heterogeneity and immune escape in personalized precision immunotherapy

The complexity of tumors arises from their multilayered heterogeneity, manifesting as spatial heterogeneity, temporal heterogeneity, and genomic heterogeneity (29, 35). Spatial heterogeneity refers to differences in gene expression, metabolic states, and treatment sensitivities among different regions of the same tumor. Temporal heterogeneity reflects the continuous evolution of new clonal subpopulations in response to growth, metastasis, and therapeutic pressures. Genomic heterogeneity stems from the genomic instability of cancer cells, presenting as high-frequency mutations, gene amplifications, and deletions. These heterogeneity factors lead to markedly different responses to therapies targeting the same biomolecule among different patients, different lesions, or even different sites within the same lesion (30, 36).

Concurrently, the mechanisms by which tumors evade immune surveillance are becoming increasingly complex. On one hand, tumor cells can directly inhibit T cell activity by upregulating immune checkpoint molecules such as PD-L1 and CTLA-4 ligands. On the other hand, the tumor microenvironment is often enriched with immunosuppressive factors (e.g., TGF-β, IL-10) and immunosuppressive cells (e.g., regulatory T cells, myeloid-derived suppressor cells, and M2 macrophages), forming a multifaceted inhibitory network. Furthermore, defects in antigen presentation or decreased antigen diversity hinder effector T cells from accurately recognizing and eliminating cancer cells (32, 34, 38, 39). The cumulative effects of these factors result in significant variability in the efficacy of identical immunotherapeutic approaches between different patients and even among different lesions within the same patient, complicating the development of reproducible personalized treatment strategies (37, 40). Thus, establishing high-fidelity models that can simultaneously reproduce the polyclonal heterogeneity of tumors, their spatiotemporal dynamics, and complex immune interactions has become a core requirement in the research of precision immunotherapy. This also indicates the direction for further refinement and application of tumor organoid technology.

It is precisely the severe challenges posed by the aforementioned tumor heterogeneity and immune escape mechanisms to individualized therapy that underscore the urgency and unique value of developing tumor organoid technology. While traditional models struggle to recapitulate such a complex microenvironment characterized by polyclonal coexistence, dynamic evolution, and profound immunosuppression, tumor organoid technology—by virtue of its three-dimensional architecture, patient-derived nature, and immune co-culture systems—offers a means to simultaneously replicate and study these challenges *in vitro*. Although current organoid models still face limitations in areas such as vascularization, long-term immune cell survival, and standardization, they serve as a critical window into understanding tumor complexity and remain an indispensable bridge for overcoming these challenges and advancing precision immunotherapy toward clinical application.

## 3 Tumor organoid model construction: from sample collection to system optimization

**Figure 2:**
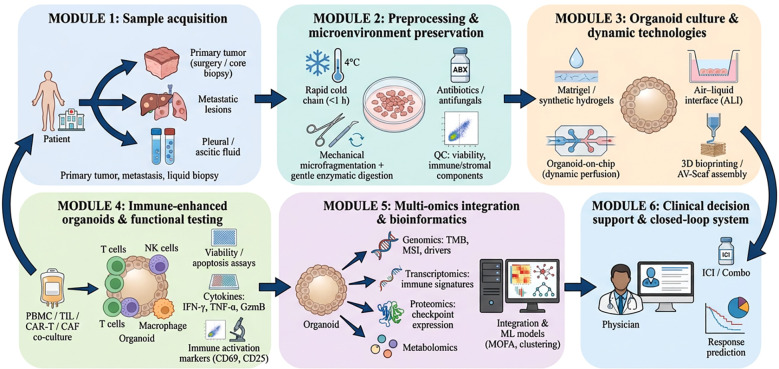
Workflow of tumor organoid technology supporting precision immunotherapy.

To provide an integrated overview of the methodology discussed in this section, **Figure 2** summarizes the complete workflow of tumor organoid construction, immune enhancement, multi-omics profiling, and clinical decision support.

### 3.1 Sample acquisition and preprocessing

The diversity of sample sources significantly determines the ability of established models to accurately reflect tumor heterogeneity and the immune microenvironment (56). Generally, there are three main sources: primary tumor tissue, metastatic lesion tissue, and liquid biopsy samples (e.g., pleural effusion, ascites). Primary tumor tissue can be obtained through surgical resection or image-guided biopsy (e.g., ultrasound or CT-guided core needle biopsy) (42, 57). Surgical specimens can provide large tissue blocks, often several centimeters in size, suitable for large-scale organoid culture. Biopsies are particularly useful for patients who cannot undergo surgical resection due to advanced disease or insufficient functional reserve. To ensure sample representativeness and viability, it is recommended to transport samples rapidly under cold chain conditions: placing tumor tissue in pre-cooled sterile culture medium (e.g., DMEM/F12 mixed base containing penicillin/streptomycin and antifungal agents) and transferring to the laboratory within 1 hour at 4 °C. Tumor metastatic lesions (e.g., lymph nodes, liver, lung, bone) are also important sources for models, as they often reflect clonal selection and immune evasion characteristics during tumor progression (12, 58). Typical collection methods include image-guided organ biopsies (CT/ultrasound-guided), surgical or endoscopic excision of lesions, and percutaneous vertebral biopsy (for bone metastases). Similar to primary tissues, sampling from metastatic lesions requires strict aseptic techniques and cold chain transport to minimize the loss of tumor stroma and infiltrating immune cells.

Pleural or ascitic fluids often contain shed tumor cells, tumor-associated macrophages, lymphocytes, etc. These samples can be obtained non-invasively or with minimal invasiveness via thoracentesis or paracentesis (59, 60). Liquid biopsies offer advantages such as non-invasiveness and the ability to perform repeat sampling to monitor tumor dynamics; however, several points must be considered: a. A sample volume of 10–50 mL is generally recommended to ensure sufficient cell numbers; b. Rapid centrifugation (300–500 g, 5–10 min) is necessary to collect cell aggregates and remove blood cell contaminants using red blood cell lysis solution; c. Mechanical shear should be minimized during processing to avoid cell rupture and the loss of surface molecules. Parallel collection of multi-modal samples can provide a more comprehensive reflection of tumor heterogeneity and differences in the immune microenvironment within the same patient. Prior to tissue submission, on-site cytological smears can be prepared for quick assessment to filter out non-tumor components, ensuring that only confirmed tumor regions are used for organoid culture (61). In a pilot study, a team assessed 64 samples on-site, achieving diagnostic specimens in 82.2% of cases, thereby providing a precise histological guarantee for subsequent tumor organoid cultures (61).

### 3.2 Organoid culture systems and dynamic technologies

#### 3.2.1 Tumor tissue pretreatment

In the sample collection and preliminary processing stages, improper handling can lead to the loss of tumor-infiltrating immune cells and stromal components, negatively impacting the ability of organoid models to accurately recreate the tumor microenvironment (62, 63). Key considerations include:(1)Cold Chain and Time Management:Samples should be placed in a 4°C culture medium immediately after collection, with the entire transport and pre-processing process ideally kept within 1 hour. Prolonged exposure to room temperature should be avoided to minimize cell apoptosis and pro-inflammatory responses. Additionally, adding 0.5% or more bovine serum albumin and antioxidants (e.g., N-acetylcysteine) to the culture medium can help buffer cellular stress (62).(2)Antibiotic and Enzyme Treatment:To reduce the risk of bacterial and fungal contamination, broad-spectrum antibiotics and antifungal agents should be added to the transport and processing media. Proteinase inhibitors (e.g., PMSF) and metalloproteinase inhibitors (e.g., EDTA or EGTA) should also be included to protect the structural integrity of matrix proteins (47).(3)Mechanical and Chemical Approaches:Combining appropriate mechanical cutting (e.g., using sterile surgical blades) with gentle enzymatic digestion (using an enzyme solution containing 0.2–1 mg/mL collagenase IV and 0.5 mg/mL dispase, incubated at 37 °C for 20–30 minutes) can help preserve the integrity of cell aggregates while maintaining the cell-matrix interaction interface.(4)Microenvironment Percentage Indicators:After pre-processing, immediate flow cytometry or immunohistochemical assessments should be conducted to evaluate the relative abundance of CD45+ immune cells, α-SMA+ fibroblasts, and collagen I/III matrix components. If the matrix components are below 50% of the *in situ* levels, it is advisable to reduce the digestion time or use lower concentrations of digestive enzymes to maintain the tumor-stroma tissue complex (50).(5)Mechanical Micro-Cutting Tools:The recent introduction of micro-instruments (e.g., µDicer and µGrater) has significantly improved the reproducibility and efficiency of mechanical cutting. These devices can generate 200–400 µm diameter tissue fragments within minutes, while effectively preserving infiltrating immune cells and matrix components (64).

Tissue preprocessing is the primary step in constructing organoids, and its quality directly influences subsequent amplification efficiency and model consistency (65). Traditional methods often rely on high concentrations of enzymes for rapid single-cell suspension generation, which can disrupt cell–cell and cell–matrix interactions, making it difficult to preserve the *in situ* structure of immune infiltrates and tumor stem cell subpopulations. Purely mechanical cutting is time-consuming and lacks reproducibility (66). Current optimized approaches typically combine the advantages of both methods, with specific steps as follows:(1)Mechanical Pre-Cutting: a. In a sterile laminar flow hood, place the cold-chain transported tissue in pre-cooled culture medium and cut with a sterile surgical blade along the direction of vascular distribution to obtain approximately 1 mm³ tissue chunks;b. If conditions permit, use a µDicer for overall homogenization of the tissue chunks. This device features a precision micro-blade array that can quickly generate uniform fragments while controlling pressure and cutting depth (66).(2)Gentle Enzymatic Digestion:a. Place the mechanically cut tissue in a digestion solution containing 0.2–0.5 mg/mL collagenase IV and 0.5 mg/mL dispase, and incubate gently in a 37°C water bath for 15–30 minutes;b. During digestion, gently pipette or invert the tubes every 5 minutes to promote the separation of the intermediate layer while avoiding excessive shear;c. After digestion, immediately add an equal volume of culture medium containing 10% fetal bovine serum to terminate enzyme activity, and filter through a 100 µm mesh to collect 200–400 µm tissue fragments (65).(3)Quality Control: a. Take a small amount of the digested fragments for live cell staining (e.g., Calcein-AM/PI dual staining) to assess cell viability, which should exceed 80%;b. Detect the distribution ratios of stem cell and matrix-related markers (e.g., LGR5, CD44, α-SMA) in the tissue fragments using flow cytometry or immunofluorescence for rapid assessment. By employing the “mechanical micro-cutting + gentle enzymatic digestion” workflow, it is possible to enhance preparation efficiency and reproducibility while ensuring cell viability and functional diversity, thus better preserving the spatial distribution of tumors along with their infiltrating immune and stromal components.

#### 3.2.2 Culture medium systems and matrix selection

The self-organization and long-term maintenance of organoid models depend on suitable base materials and combinations of growth factors. The commonly used systems include the following:(1)Matrigel is rich in laminin and collagen IV, typically used at concentrations of 8–10 mg/mL. It is embedded as 50 µL droplets in 24-well plates to form semi-solid droplets. After solidification, a complete culture medium based on DMEM/F12 is added, commonly including EGF (50 ng/mL), R-spondin 1 (500 ng/mL), Wnt3A conditioned medium (10–15%), Noggin (100–200 ng/mL), and additives like N2/B27, insulin, and selenium. This system can effectively support the three-dimensional growth, redifferentiation, and cryopreservation of organoids, making it suitable for most solid tumor-derived organoid cultures (67–69).(2)To achieve controlled composition and batch consistency, recent years have seen the development of PEG-based, alginate-based, and GelMA modified hydrogels. These materials allow for the modulation of matrix stiffness (in the range of 0.5–2 kPa) through crosslinking, facilitating studies on the impact of matrix mechanics on tumor cell proliferation and signaling pathway activation. Compared to Matrigel, synthetic hydrogels offer advantages such as lower immunogenicity, the ability to introduce specific adhesive peptide sequences (e.g., RGD), and ease of chemical modification and high-throughput automated preparation (70–73).(3)In Air-Liquid Interface (ALI) culture, tissue fragments or organoids are embedded in collagen I or Matrigel and placed above a Transwell plate with a semi-permeable membrane. The lower chamber is filled with complete culture medium, ensuring continuous immersion of the base while maintaining a humidified gas phase above. This strategy significantly improves oxygen diffusion efficiency and preserves the inherent stroma and immune cells of the tumor. Tumor organoids generated through ALI culture can co-express tumor cells, fibroblasts, and various infiltrating immune subpopulations over extended periods, facilitating immune drug screening without the need for additional reconstruction (74–77).

#### 3.2.3 Dynamic culture systems and applications of emerging technologies

Static culture conditions fail to simulate the continuous supply of nutrients and the removal of metabolic waste found *in vivo*, often leading to central hypoxia or nutrient unevenness. The integration of dynamic culture and emerging technologies provides new strategies for constructing a more physiologically relevant tumor microenvironment (78).(1)Microfluidic Chips (Organoid-on-Chip):This technology introduces organoids or tumor cells along with the matrix into a network of microchannels. By precisely designing flow rates and shear force gradients, it enables the dynamic delivery of nutrients, oxygen, and cytokines while facilitating waste metabolism. This approach allows for quantitative assessments of drug transport kinetics, cell migration pathways, and real-time interactions between immune and tumor cells (45, 78–80).(2)3D Bioprinting:Utilizing low-shear extrusion or digital light processing techniques, tumor cells, stromal cells, and pre-fabricated bioinks (including collagen, hyaluronic acid, and gelatin methacrylate) are layered to create complex structures. 3D bioprinting not only forms highly spatially resolved scaffold architectures but also allows for the precise localization of different cell subpopulations, achieving accurate recapitulation of heterogeneous tissues. Recent studies indicate that using patient-derived cells for 3D bioprinting can swiftly generate *in vitro* immune-tolerant or susceptible models, providing a new platform for investigating mechanisms of immune therapy resistance (81–84).(3)Acoustic Virtual Platforms without Scaffolds (AV-Scaf):This method utilizes acoustic fields to drive cells to self-assemble in three-dimensional space, forming size-controlled aggregates without the need for exogenous scaffold materials. This technique accelerates organoid formation and facilitates direct contact between tumor and immune cells, significantly enhancing T cell activation states, as evidenced by substantial upregulation of Granzyme B and IFN-γ, thereby aiding efficient assessments of immune responses (85). By combining these various technologies, dynamic culture systems can more authentically reconstruct the characteristics of tumors *in vivo*, providing a more reliable *in vitro* platform for precision immunotherapy research.

### 3.3 Immune co-culture and functional enhancement

While conventional tumor organoids can retain certain tumor cell heterogeneities, they often lack immune components, making it challenging to comprehensively assess immunotherapeutic effects. Therefore, constructing “immune-enhanced” organoids has become a focal point in research.(1)Retention of Autologous Infiltrating Immune Cells:Immune cells such as tumor-infiltrating lymphocytes (TILs) and macrophages retained through ALI or micro-cutting technologies (µDicer/µGrater) can participate in immune response assessments without additional reconstruction. This model has been successfully applied in studies of anti-PD-1 immunotherapy responses, demonstrating immune cell compositions and functional characteristics similar to those found *in vivo* (64, 86).(2)Reconstruction of Exogenous Immune Cells:a. PBMC Co-Culture: Peripheral blood mononuclear cells (PBMCs) can be seeded onto the surface of organoids or in suspension at ratios of 5:1 to 20:1, with culture medium supplemented with IL-2 and IL-7 to promote T cell expansion and activation (87);b. TIL Reconstruction: High-purity CD8+ TILs can be obtained through Raman sorting or microfluidic sorting and then directed into tumor organoids to study tumor-specific immune recognition and killing mechanisms;c. Involvement of CAFs: Introducing patient-derived cancer-associated fibroblasts (CAFs) can create a ternary co-culture system that includes intrinsic immune cells, tumor cells, and stromal cells. This setup better simulates the immunosuppressive microenvironment and the impact of fibrotic barriers on immune cell infiltration (88).(3)Functional Interaction Verification:Real-time imaging systems (IncuCyte) can be used to monitor T cell aggregation and cytotoxicity against organoids. Flow cytometry can assess the expression levels of cytokines like Granzyme B and IFN-γ to quantitatively evaluate immune-mediated cytotoxic responses. In co-cultured melanoma organoids using the AV-Scaf platform, T cell expression of Granzyme B increased from 2.82% to 17.5%, and IFN-γ from 1.36% to 16%, indicating a significant enhancement in immune activity (85).

To elucidate mechanisms of immune evasion and optimize immunotherapy, gene editing and functional marking techniques can be introduced into tumor organoids. The CRISPR-Cas9 system can be utilized to target and knock out or activate key immune checkpoint genes (such as PD-L1, CTLA-4, TIM-3, etc.). Monoclonal screening can then be employed to obtain gene knockout or overexpression clones. After gene editing, comparisons can be made within the same batch of organoids regarding immune cell infiltration and cytotoxicity under different gene statuses, aiding in the identification of potential therapeutic targets or resistance mechanisms (89). Fluorescent proteins (e.g., GFP, mCherry, ZsGreen) or reporter genes (e.g., firefly luciferase) can be transduced into different cell subpopulations to enable multicolor tracking of tumor cells, fibroblasts, and T cells. Coupled with real-time imaging and high-content analysis, this approach allows for precise quantification of the spatiotemporal distribution and dynamic interactions among various subpopulations (90). Applying single-cell RNA sequencing (scRNA-seq) and single-cell ATAC sequencing (scATAC-seq) to organoid samples helps characterize the weighted features of immune cells, tumor stem cell subpopulations, cytokine networks, and signaling pathway activation states. For example, by performing scRNA-seq on cells at different time points in co-culture models, researchers can track T cell activation, exhaustion, and memory processes while identifying key regulatory factors (91).

To enhance the reproducibility and data utility of organoid models, a comprehensive data management and analysis platform needs to be established.(1)Data Standardization and Quality Control:A unified metadata template should be created, including patient information, sample collection methods, culture parameters, and batch numbers. This data can be recorded and tracked in a Laboratory Information Management System (LIMS) in real-time. Key quality control (QC) indicators should be established, such as tissue fragment size distribution, cell viability, marker expression, and genomic stability, and verified before and after each culture batch.(2)Bioinformatics Support:Open-source software tools (such as Seurat and Scanpy) can be used for the processing and visualization of single-cell omics data. Tumor-immune interaction network models can be constructed, utilizing cell communication analysis tools (like CellChat and NicheNet) to infer potential ligand-receptor pairs. Moreover, machine learning and deep learning algorithms can be applied to perform integrative analyses of multi-omics data (genomics, transcriptomics, proteomics, metabolomics, etc.) to discover biomarkers associated with responses to immunotherapy or drug resistance.(3)Multi-Center Collaboration and Open Resources:Patent and literature trend analyses indicate that over 700 patents related to organoid technology have been filed globally, with a significant increase in applications since 2015, particularly in tumor model construction and drug screening, accounting for 76.3% of the patents (92). This provides commercial and technical support for establishing an international, multi-institutional database for tumor organoids and immunotherapy. Laboratories should be encouraged to use SOPs for cross-validation. Additionally, raw and processed multi-omics data should be uploaded to data-sharing platforms to facilitate algorithm iteration and model optimization. Furthermore, a biorepository should be jointly established to deeply link original organoid materials with detailed culture parameters and response data.

Through these multidisciplinary optimization strategies, the support capability of tumor organoid models for precision immunotherapy research can be continually enhanced, providing a solid data and technical foundation for clinical translation.

### 3.4 Technical variations and unique challenges in co-culture with different immune cells

The central challenge associated with T cells stems from their reliance on persistent antigen-specific activation and clonal expansion. Technically, this necessitates either co-culture systems (e.g., with antigen-presenting cells) or genetic engineering approaches (such as CAR-T construction) to provide primary and secondary activation signals. Consequently, the principal challenge lies in maintaining T cells’ functional activation, clonal expansion capacity, and cytotoxic efficacy over extended periods within 3D organoids, while effectively preventing functional exhaustion.

The uniqueness of the challenge with macrophages is rooted in their high contextual plasticity. A key technical distinction is that macrophages do not require antigen-specific pre-sensitization; however, precise regulation of microenvironmental cues (e.g., GM-CSF, IFN-γ vs. M-CSF, IL-4/IL-13) is essential to direct their phenotypic polarization. Thus, the major challenge involves accurately steering and stabilizing their polarization toward the anti-tumor M1 phenotype within co-culture systems, while preventing their “reprogramming” by the tumor microenvironment into the pro-tumorigenic M2 phenotype.

​ Challenges with NK cells are primarily linked to their rapid response mechanism, MHC-unrestricted killing, and relatively short *in vivo* lifespan. Technically, maintaining NK cell survival and basal activity relies more heavily on cytokine support (e.g., IL-15) rather than complex pre-activation protocols. The distinctive challenge, therefore, centers on overcoming their rapid functional decline *in vitro*. This involves optimizing cytokine cocktails to counteract immune evasion mechanisms—such as tumor cell upregulation of HLA-I molecules or other resistance pathways—that may compromise NK cell recognition and cytotoxicity.

## 4 Precision immunotherapy applications: from drug screening to mechanism elucidation

This chapter summarizes the application value of tumor organoid models in precision immunotherapy, focusing on organoid-based immunotherapy drug screening platforms, the interaction mechanisms between immune cells and tumor cells, and how organoids can support personalized immunotherapy. The following sections detail the technical highlights, application cases, and future development directions in each subfield.

### 4.1 Organoid-based immunotherapy drug screening

In recent years, as tumor immunotherapy has become a clinical hotspot, traditional two-dimensional cell culture models and animal experiments have shown significant limitations in simulating the real tumor microenvironment and tumor heterogeneity. Tumor organoid models, which effectively retain the tissue structure, intercellular interactions, and some *in situ* immune components of primary tumors, have gradually become important platforms for screening immune-related drugs (93). In the study of immune checkpoint inhibitors (ICIs), multiple experiments focusing on colorectal cancer, breast cancer, and non-small cell lung cancer have utilized organoid systems to observe T cell activation and cytokine release (94). Researchers have constructed patient-derived tumor organoid and exogenous immune cell co-culture systems that not only simulate the inhibitory effects of the PD-1/PD-L1 signaling pathway but also enable accurate quantitative assessments of ICI efficacy by detecting T cell infiltration and cytotoxicity indicators within the organoids (95). Additionally, the application of bispecific antibodies (BiTEs) has been well validated using organoid platforms. These drugs can simultaneously bind to tumor-associated antigens and T cell receptors, promoting T cell accumulation near tumor cells, thereby significantly enhancing tumor cell killing efficiency. By incorporating specific markers in the organoid co-culture system, researchers can observe the direct contacts and killing effects between T cells and tumor cells in real-time, reflecting the biological effects produced after drug-mediated immune cell activation (96). Furthermore, the development of personalized tumor vaccines based on organoids has become a research hotspot in this field (97, 98). Utilizing organoid models, researchers can screen and predict neoantigens expressed in patient tumors and subsequently test the activation effects of immune vaccines *in vitro*. This method not only ensures that tumor heterogeneity is fully considered during the screening process but also simulates the real conditions of the patient’s original tumor microenvironment in immune response evaluations, providing data support for further personalized vaccine strategies (99). In these studies, the secretion of immune cytokines, such as IFN-γ and TNF-α, serves as important reference indicators for evaluating drug effects, providing experimental evidence for optimizing clinical immunotherapy. In summary, the drug screening platforms constructed using organoids, leveraging their ability to retain native tumor heterogeneity and local immune environments, have demonstrated immense potential in the screening of ICIs, bispecific antibodies, and personalized vaccines, laying a solid foundation for immunotherapy drug development and clinical translation.

In the tumor organoid platform, constructing precise drug response profiles is crucial for evaluating the sensitivity and resistance of immunotherapy drugs. First, methods such as MTT and CCK-8 assays can quantitatively assess the survival rates of organoid cells following drug treatment, providing intuitive evidence for preliminary drug effect screening. Concurrently, techniques like Annexin V/PI staining and caspase-3 activation detection can monitor drug-induced apoptosis, revealing the molecular mechanisms underlying the cytotoxic effects on tumor cells (100). In terms of immune effects, the organoid platform often evaluates immune cell-mediated cytotoxic responses by measuring the expression levels of perforin, Granzyme B, IFN-γ, and TNF-α (101). This multiparameter detection approach can eliminate potential random errors associated with single indicators, enhancing the sensitivity and accuracy of drug screening. For instance, in the same patient-derived organoid system, the quantitative changes in cytokine levels after different drug treatments often exhibit significant individual variability. This heterogeneity reflects the complex regulatory mechanisms of drug responses at genetic and epigenetic levels (100).

To visually present drug response profiles, some studies employ multidimensional data analysis techniques, such as heatmaps and radar charts, to compare the activity, apoptosis rates, and immune cell activation of different drugs within organoid models (102). Such data integration not only aids in identifying potential efficient drug combination strategies but also provides rich experimental evidence for further exploration of tumor resistance mechanisms. By comparing organoids derived from different patients, insights can be gained into the sensitivity of certain drugs to specific molecular subtypes, laying a theoretical foundation for precision personalized medicine (103). Overall, the multiparameter detection strategy and rigorous quantitative assessment methods enable the organoid platform to construct drug response profiles that reflect both cell viability and apoptosis, while accurately capturing the dynamic changes in the release of specific cytokines by immune cells. This provides reliable experimental support for the preliminary screening of immunotherapy drugs and clinical prognosis.

### 4.2 Mechanistic insights into tumor–immune interactions

To deeply explore the molecular mechanisms of tumor immunotherapy and the principles of immune evasion, constructing co-culture systems of organoids and immune cells has become essential (104). In these systems, patient-derived PBMCs, TILs, or DCs are typically used to replicate the interactions between immune cells and tumor cells within the tumor microenvironment (105). In specific experimental designs, the timing and ratio of immune cell addition are critical. Studies have shown that introducing immune cells during the early stages of organoid formation or after the tumor cells have partially established a 3D structure significantly impacts T cell activation status, cell migration, and tumor cell killing efficacy. Notably, the activation state and infiltration depth of T cells directly determine the efficiency of apoptosis and lysis within the organoids. Techniques such as flow cytometry and immunofluorescence staining can accurately monitor the expression of T cell surface activation markers (e.g., CD69, CD25) and cytokine secretion, thereby dynamically reflecting the balance between immune cell activity and tumor cell resistance (106). Additionally, tumor-associated macrophages (TAMs) play a crucial role in co-culture models. Research indicates that TAMs can polarize into M1 (pro-inflammatory, anti-tumor) or M2 (immunosuppressive, pro-tumor) phenotypes within the tumor microenvironment. The distinct functions of different macrophage subtypes in regulating tumor cell invasion and immune responses are evident. By finely tuning the ratio of macrophages in the co-culture system, researchers can observe the dynamic balance between immune subpopulations and their regulatory effects on tumor cell growth curves, providing direct experimental evidence for elucidating immune escape mechanisms (107, 108). Moreover, utilizing multispectral imaging and high-resolution confocal microscopy allows for real-time tracking of immune cell spatial distribution and migration paths within organoids, further elucidating the direct contacts and signaling processes between tumor cells and various immune cell types. This dynamic interaction research not only aids in understanding how immune cells exert synergistic cytotoxic effects in the tumor microenvironment but also provides a theoretical basis for optimizing immunotherapy strategies and enhancing treatment sensitivity (93).

In the application of tumor immunotherapy, elucidating the complex regulatory relationships of cell signaling and cytokine networks is key to revealing the mechanisms of immunotherapy. Currently, various molecular biology techniques are widely utilized in this field, including Western blotting, quantitative real-time PCR (qPCR), and enzyme-linked immunosorbent assays (ELISA). These methods effectively detect the activation states of PD-L1, IFN-γ downstream regulatory factors, and other key signaling molecules, allowing for quantitative assessment of molecular changes following immunotherapeutic interventions (109). Simultaneously, multiplex fluorescent staining combined with confocal microscopy can visually demonstrate direct interactions between tumor cells and immune cells, such as the formation of immunological synapses and the distribution of intercellular signaling points. These imaging techniques not only provide precise localization for studying intracellular signaling but also offer dynamic imaging support for real-time monitoring of T cell activity, migration, and cytotoxic processes. The recent introduction of live-cell imaging systems has significantly advanced the study of spatiotemporal dynamics, enabling researchers to observe T cells as they migrate within organoids, recognize, and kill tumor cells in real-time (109, 110). Furthermore, cytokine network regulation plays a crucial role in modulating immune balance within the tumor microenvironment. By analyzing signaling pathways such as IL-6/STAT3 and Th1/Th2 balance, researchers can uncover the complex exchange mechanisms between immune and tumor cells. As artificial intelligence and machine learning technologies continue to mature, image recognition and automated data processing have become vital supplementary tools for real-time imaging techniques. This not only enhances data processing efficiency but also greatly expands the depth of analysis regarding complex signaling events (111, 112). While there are still certain limitations in interpreting specific cell communication events, the future integration of multimodal imaging with automated algorithms will undoubtedly provide more robust technical support for a comprehensive understanding of immunotherapy mechanisms.

### 4.3 Personalized immunotherapy and multi-omics integration

The establishment of personalized treatment prediction systems aims to move away from traditional “one-size-fits-all” treatment models, tailoring the most suitable immunotherapy strategies for each patient. Patient-derived tumor organoids serve as an ideal platform for predicting immunotherapy outcomes due to their ability to preserve the molecular characteristics and complex microenvironment of tumors in their native state. In this system, by simulating immune checkpoint inhibition, CAR-T cell therapy, and other immunotherapeutic interventions *in vitro*, researchers can comprehensively assess the effects of various treatment strategies on tumor growth in patients (113, 114). Specifically, tumor samples obtained from patient surgeries or biopsies undergo processing to create 3D organoid models that retain complete immune components. Subsequently, by applying different immunotherapy drugs to these organoids, researchers can observe key indicators such as T cell activation, cytokine release, and tumor cell apoptosis that may occur *in vivo*. Quantitative detection of this data, when compared with the patient’s clinical responses, can accurately predict treatment response rates and the likelihood of adverse events, thereby providing substantial support for clinicians in treatment selection and dynamic efficacy assessment (96, 100). This “patient-organoid-clinical feedback” prediction system not only reflects tumor heterogeneity at the individual level but also enables real-time adjustments to treatment strategies during immunotherapy. In some prospective cohort studies, the sensitivity to immunotherapeutic agents detected by organoid models has shown a high correlation with the eventual treatment outcomes in patients, providing precise evidence for subsequent clinical interventions (115, 116). By leveraging this platform, continuously optimizing treatment processes and drug combinations holds the promise of significantly enhancing overall response rates to immunotherapy while reducing the risks of ineffective treatments and unnecessary adverse reactions.

Within the framework of precision medicine, the systematic integration of multi-level molecular information—including genomic, transcriptomic, and proteomic data—is crucial for advancing personalized immunotherapy. Tumor organoids, which preserve the heterogeneity and microenvironmental features of the original tumors, serve as an ideal platform for such multi-omics integration. Current strategies primarily adopt a “hierarchical complementarity, function-oriented” analytical framework, fusing data from different sources and dimensions to collectively optimize immunotherapy decision-making. The specific integration approaches operate at the following levels. Integrated analysis of genomic and transcriptomic data begins with identifying tumor mutational burden (TMB), microsatellite instability (MSI) status, and specific gene mutations (e.g., in *POLE/POLD1*) from whole-genome or exome sequencing data of organoids. These genomic findings are then correlated with transcriptomic sequencing (RNA-seq) data to analyze the expression of mutation-derived neoantigens and evaluate T-cell inflammation gene expression signatures. This combined analysis provides a comprehensive assessment of the tumor’s immunogenic potential (117). Functional validation and complementarity between transcriptomic and proteomic data involves leveraging transcriptomic data to reveal immune cell infiltration patterns and mRNA expression levels of immune checkpoint molecules (e.g., PD-L1, CTLA-4), while recognizing that proteins are the ultimate functional executors. It is therefore essential to validate the actual expression and spatial localization of key immune molecules at the protein level using techniques such as flow cytometry, CyTOF (mass cytometry), or immunohistochemistry. This cross-omics validation helps to circumvent discrepancies caused by post-transcriptional regulation, thereby enhancing the reliability of biomarker identification (118).Multi-omics data integration and modeling begins with quality control and standardization of multi-dimensional data, after which various computational methods can be employed for integration. Multi-Omics Factor Analysis (MOFA), for example, can be used to extract common sources of variation across omics layers, thereby identifying core molecular modules that drive immune phenotypes. Alternatively, machine learning algorithms such as random forest or support vector machines can be utilized to construct predictive models with drug response or immune phenotypes as outputs. Input features may include gene mutation profiles, pathway activity scores, immune cell composition, and key protein expression levels, enabling precise mapping from multi-dimensional data to treatment response (117).

In practical implementation, systematic multi-omics profiling is first performed on tumor organoids. The integrated strategies described above are then applied to classify patient-derived organoids into distinct immune functional subtypes, such as “hot tumors,” “immune-excluded,” and “immune-desert” phenotypes. Based on this refined subtyping, combined with an AI-driven drug screening platform, researchers can identify patient populations most likely to respond to immune checkpoint inhibitors, adoptive cell therapy, or other combination regimens. This approach also provides clues for overcoming drug resistance through rational combination strategies. Furthermore, the construction of an integrated “organoid–multi-omics data–clinical decision” platform serves as a critical vehicle for translating integrated data into clinical practice. This platform links multi-omics analysis results with clinical knowledge bases and drug databases. Through intelligent recommendation algorithms, it provides clinicians with decision support—including immunotherapy selection, combination therapy suggestions, and efficacy prediction—forming a closed-loop management system from molecular profiling to treatment implementation (118, 119).

## 5 Progress in application: multi-cancer cases and new drug development

### 5.1 Case studies and translational progress in major cancer types

In colorectal cancer (CRC) research, patient-derived organoids (PDOs) have emerged as a vital model that can replicate the heterogeneity of primary tumors (120–122). The choice of sampling strategy is crucial, as both primary and metastatic sites can serve as sources. Researchers often compare and analyze differences in gene expression and drug sensitivity between these two sources to gain a more comprehensive understanding of tumor biology (122, 123). The optimization of culture media is a key step in PDOs construction. The regulation of the Wnt signaling pathway plays a central role in maintaining colorectal stem cell characteristics and long-term growth of organoids. Hence, growth factors such as Wnt3a and R-spondin1 are commonly added to the culture medium to activate the Wnt signaling pathway, promoting organoid formation and expansion. Moreover, studies have indicated that CRC PDOs hold potential for predicting responses to immunotherapy. By assessing the response of PDOs to PD-1/PD-L1 inhibitors, researchers can predict patient sensitivity to immune checkpoint inhibitors, thereby guiding clinical treatment decisions (122).

Breast cancer is a highly heterogeneous disease, classified into different subtypes based on hormone receptor (ER, PR) and HER2 expression, including hormone receptor-positive, HER2-positive, and triple-negative breast cancer (TNBC) (124). Organoid models play a significant role in drug screening and mechanistic studies for the various subtypes of breast cancer. Hormone receptor-positive breast cancer organoids can be used to study mechanisms of resistance to endocrine therapies and to screen for new targeted drugs. HER2-positive breast cancer organoids are utilized to evaluate the efficacy of anti-HER2 targeted therapies. In contrast, TNBC organoids, which lack clear therapeutic targets, are often employed in immunotherapy and new drug development (125, 126). For instance, researchers can use TNBC organoids to screen for PARP inhibitors or assess the anti-tumor activity of CAR-T cell therapies (127–129). In metastatic breast cancer, studies have shown that tumor cells cultured from malignant pleural effusion (MPE) can be used to construct organoids. Co-culturing these patient-derived organoids with AdCAR-T cells or conducting drug sensitivity testing with biotinylated monoclonal antibodies targeting CD276, HER2, EGFR, TROP2, or EpCAM has revealed that AdCAR-T cells exhibit specific organoid-killing effects based on individual antigen expression patterns (129).

Non-small cell lung cancer (NSCLC) is the most common type of lung cancer, characterized by a complex spectrum of driver gene mutations, including EGFR mutations and ALK fusions (130). Researchers have constructed corresponding organoid models based on different driver gene backgrounds and applied them to assess sensitivity to immune checkpoint inhibitors (131, 132). For NSCLC organoids with EGFR mutations, researchers evaluate their response to EGFR-TKI drugs and explore mechanisms of resistance. In the case of ALK fusion NSCLC organoids, sensitivity to ALK inhibitors is assessed. Additionally, PD-L1 expression levels are crucial indicators for predicting the efficacy of immunotherapy in NSCLC. Studies have shown that NSCLC organoids with high PD-L1 expression exhibit greater sensitivity to immune checkpoint inhibitors, such as anti-PD-1 monoclonal antibodies (131, 133).

Gastric cancer is one of the most common malignant tumors globally, with its development linked to various factors, including Helicobacter pylori infection and dietary habits (134–136). The construction of gastric cancer organoids provides a novel platform for studying the mechanisms of gastric cancer development, drug screening, and personalized treatment (137, 138). Research indicates that gastric cancer organoids can simulate the onset and progression of gastric cancer and are utilized for screening and developing anti-tumor drugs, as well as for personalized and targeted therapies (139). Suspension culture methods have successfully constructed gastric cancer organoids that closely resemble original tissues, serving as effective models for personalized drug screening (140).

In addition to the aforementioned cancers, organoid technology has been applied in studies of pancreatic cancer, liver cancer, melanoma, ovarian cancer, and more (140–148). There are differences in culture conditions across different cancer types, such as the choice of matrix and the requirements for growth factors. For example, constructing pancreatic cancer organoids typically requires the addition of growth factors like epidermal growth factor (EGF) and fibroblast growth factor (FGF) to promote organoid growth and differentiation. Liver cancer organoids are more sensitive to hypoxic environments and need to be cultured under low-oxygen conditions. Patient-derived melanoma organoid (MPDO) models offer promising avenues for understanding tumor complexity, validating treatment strategies, and potentially advancing personalized therapy. The application of tumor organoid models in immunotherapy is detailed in **Table 2**.

**Table 2:** Application cases and clinical relevance of tumor organoid models in immunotherapy drug screening.

Cancer type	Immunotherapy drug/approach	Organoid model construction method	Key detection metrics	Clinical relevance	References
Colorectal Cancer (MSI-H)	PD-1 inhibitor (e.g., Nivolumab)	Primary tumor organoids + autologous TIL co-culture	T-cell infiltration rate, IFN-γ secretion, tumor cell apoptosis rate	Predictive of patient pCR (pathological complete response)	(120–123)
Triple-Negative Breast Cancer	CAR-T cells (targeting CD276/HER2)	Pleural effusion-derived organoids + PBMC co-culture	Organoid lysis rate, Granzyme B expression	Improved response rate in personalized antigen-matched therapy	(125–129)
Non-Small Cell Lung Cancer	PD-L1 inhibitor (e.g., Atezolizumab)	Air-liquid interface (ALI) culture preserving native immune microenvironment	PD-L1 expression level, T-cell activation markers (CD69)	PD-L1-high organoids correlate with patient response rates	(131–133)
Gastric Cancer	Bispecific antibodies (BiTEs)	Surgical sample-derived organoids + exogenous T-cell reconstitution	Immune synapse formation, tumor cell survival rate	Successful screening of alternative regimens for resistant patients	(137–140)

There are significant differences in the sensitivity of various cancers to immunotherapy, influenced by factors such as genomic characteristics, TME, and the state of the immune system (128, 129, 149, 150). For instance, colorectal cancer can be categorized into microsatellite instability-high (MSI-H) and microsatellite stable (MSS) subtypes. MSI-H colorectal cancer, with a higher tumor mutation burden (TMB) and greater immunogenicity, shows a higher response rate to immune checkpoint inhibitors (122). In breast cancer, tumors can be classified as “cold” or “hot” based on the degree of immune cell infiltration. Hot tumors, which have more tumor-infiltrating lymphocytes (TILs) and higher PD-L1 expression levels, are more sensitive to immunotherapy (125, 126). In NSCLC, patients with high PD-L1 expression have higher response rates to immune checkpoint inhibitors; however, some patients with low PD-L1 expression also benefit from immunotherapy (131). The TME is a critical factor affecting the immunotherapy sensitivity of organoids (149, 150). Immunosuppressive cell populations in the TME, such as myeloid-derived suppressor cells (MDSCs), tumor-associated macrophages (TAMs), and regulatory T cells (Tregs), can inhibit immune cell activity, thereby reducing tumor sensitivity to immunotherapy (151). Components of the ECM also influence immune cell infiltration and function. To more accurately assess differences in immunotherapy sensitivity across various cancers, researchers often combine preclinical data (e.g., IFN-γ secretion levels, T cell infiltration) for analysis (149). By measuring IFN-γ secretion levels from T cells in co-culture systems, researchers can gauge T cell activation and anti-tumor activity. Additionally, using immunohistochemistry or flow cytometry to assess T cell infiltration in organoids can evaluate the tumor’s ability to attract immune cells. Multicenter studies and meta-analyses also provide essential data support for assessing differences in immunotherapy sensitivity across various cancer types (152, 153).

### 5.2 Organoid platforms: high-throughput drug screening and new drug development practices

Organoid technology has emerged as a powerful *in vitro* model, demonstrating immense potential in drug development. Compared to traditional two-dimensional cell lines and PDX models, organoids offer higher throughput, real-time monitoring, and reproducibility, making them ideal platforms for large-scale drug screening (154, 155).

Organoids can be cultured in microplates, allowing for automated processes that facilitate high-throughput screening. Techniques such as live-cell imaging and metabolic analysis enable real-time monitoring of drug effects on organoids, providing dynamic data on drug responses. Furthermore, organoids exhibit good batch-to-batch stability, ensuring the reproducibility of experimental results. The application prospects of organoids in high-throughput drug screening are extensive (154–158). Researchers can employ organoids to screen combination therapies to identify drug pairs with synergistic effects. Organoids can also be used to evaluate immunomodulators, such as immune checkpoint inhibitors and CAR-T cells. Additionally, they serve as valuable tools for predicting drug toxicity and synergistic effects, providing a basis for clinical trial design. For example, in the screening of bispecific antibodies, organoids can assess the ability to target tumor cells and activate immune cells. In oncolytic virus screening, organoids can evaluate the efficacy against tumor cells and safety for normal cells. Similarly, during tumor vaccine screening, organoids can be used to assess the capability of inducing immune responses.

To maximize the utility of organoid technology, several organoid biobanks have been established internationally, such as HUB Organoids and PDO-X (155, 159). These biobanks collect tumor samples from diverse patients and have developed standardized organoid culture protocols and quality control metrics. The establishment of these biobanks provides researchers with rich resources, facilitating the application of organoid technology in drug development and precision medicine. The standards for establishing organoid biobanks primarily include several key aspects: Diversity of Sample Sources: This includes samples from primary tumors, metastatic sites, and other relevant tissues. Integration of Clinical Data: Comprehensive datasets should encompass genomic, transcriptomic, and proteomic information. Establishment of Sharing Mechanisms: This promotes collaboration and data exchange among researchers. Through multi-omics analysis of organoids, researchers can gain deeper insights into the molecular characteristics of tumors, providing a basis for individualized treatment. Additionally, organoid biobanks should implement robust quality control systems, including morphological assessments, genomic stability testing, and functional assays, to ensure the quality and reliability of the organoids. Multi-center studies play a crucial role in driving the standardization of organoid technology and its clinical applications (160, 161). The European Organoid Consortium (UROCA) has improved data comparability by establishing standardized cultivation processes and quality control indicators, promoting the formulation of personalized treatment plans. Multi-center research can also expand sample sizes, enhancing the statistical power of studies and allowing for more accurate assessments of the value of organoids in drug development and precision medicine.

### 5.3 Clinical translation and cutting-edge applications

#### 5.3.1 Application of preclinical trial data in prognosis assessment and individualized treatment adjustments

Organoids serve as a powerful *in vitro* model with significant potential for clinical translation (66, 106, 140, 162–166). They can assist in clinical decision-making; for instance, organoid drug sensitivity testing can help identify alternative treatment options for patients exhibiting resistance. In the context of neoadjuvant immunotherapy, organoids can be used to predict efficacy, such as the pathological complete response rate (pCR).

Regarding the correlation between organoids and patient clinical outcomes, multiple studies have further validated their predictive value. For instance, Vlachogiannis et al. established a living biobank using organoids derived from patients with metastatic gastrointestinal cancer and demonstrated that *in vitro* drug sensitivity in organoids accurately predicted clinical responses, achieving a positive predictive value of 88% and a negative predictive value of 100% (66). In the context of neoadjuvant chemoradiotherapy for rectal cancer, Yao et al. conducted drug sensitivity tests on organoids derived from 80 patients with locally advanced rectal cancer. The results showed a high concordance between organoid responses to chemotherapy or radiotherapy and actual patient outcomes, with a predictive accuracy of 84.43% (162). Furthermore, Wang et al. evaluated the efficacy of pyrotinib in HER2-mutant advanced lung adenocarcinoma using both lung cancer organoids and patient-derived xenograft (PDX) models. The results aligned with subsequent Phase II clinical trial data, confirming the reliability of organoids in predicting targeted therapy response (163).Other significant studies include the work by Ooft et al., who performed irinotecan sensitivity testing on organoids derived from patients with metastatic colorectal cancer. They found a significant correlation between organoid response and clinical efficacy, providing the first prospective evidence that organoids can predict chemotherapy response in metastatic colorectal cancer (164). The Driehuis group constructed a biobank of pancreatic ductal adenocarcinoma organoids and conducted high-throughput drug screening, revealing that EZH2 inhibitors were effective against specific subtypes, with organoid drug sensitivity closely matching the genetic profiles of patient tumors (165). Additionally, Sachs et al. established a biobank comprising over 100 breast cancer organoids. When modeling HER2-targeted therapy, they observed that variations in organoid sensitivity to afatinib aligned with the molecular subtypes of the patients’ primary tumors, successfully recapitulating clinically observed targeted drug resistance phenomena (166).

Collectively, these studies—spanning gastrointestinal cancers, pancreatic cancer, breast cancer, and other tumor types—consistently demonstrate the reliability of organoids in predicting responses to chemotherapy, targeted therapy, and radiotherapy. Organoid drug sensitivity testing shows a meaningful correlation with patient progression-free survival (PFS) and overall survival (OS). As such, organoids can serve as “avatars” or surrogate models to predict individual patient treatment responses and guide clinical decision-making. For example, if an organoid shows sensitivity to a particular drug, the patient is also likely to benefit; conversely, if the organoid exhibits resistance, administration of that drug to the patient should be reconsidered.

#### 5.3.2 Prospects of combining emerging technologies with organoid platforms

The integration of organoid technology with emerging technologies such as nanomedicine, microfluidics, and 3D bioprinting presents new opportunities in tumor research and treatment (84, 132, 149, 150, 167–174).

Nanomedicines offer advantages such as superior targeting capability and high drug-loading capacity, which can enhance therapeutic efficacy and reduce systemic toxicity (151). Organoids can be utilized to evaluate the targeted delivery efficiency of nanocarriers (e.g., liposomes, exosomes). By observing the distribution of nanocarriers within organoids and monitoring drug release kinetics, their targeting specificity and effectiveness can be assessed. Furthermore, organoids serve as a robust model for evaluating the ability of nanodrugs to penetrate vascular barriers, thereby providing critical insights for the design and optimization of nanomedicine-based therapies. Recent studies have further demonstrated the potential of this integrated strategy in immunotherapy. For instance, Ni et al. (167) developed a nanoplatform composed of Hf-based metal-organic frameworks (nMOFs) loaded with an immune adjuvant and αCD47 antibody. Their organoid model revealed that these nanoparticles effectively promoted dendritic cell maturation and antigen presentation, thereby triggering a systemic anti-tumor immune response. In another study, Chen et al. (168) designed folic acid-modified manganese–protoporphyrin liposomes (FA-MnPs). A co-culture model using organoids confirmed that, upon ultrasound stimulation, these particles effectively remodeled the tumor immune microenvironment by promoting the polarization of tumor-associated macrophages toward the pro-inflammatory M1 phenotype and enhancing the infiltration of cytotoxic T cells. Additionally, Du et al. (169) reported a lipid-based nanoparticle incorporating GEM and LMWH (low molecular weight heparin). Organoid-based experiments demonstrated that this formulation effectively normalized tumor vasculature and alleviated the hypoxic microenvironment, thereby enhancing the efficacy of subsequent immunotherapy.

Microfluidic technology enables the simulation of dynamic changes in the tumor microenvironment—such as T-cell migration and cytokine gradients—on microchips (106, 132). Organ-on-a-Chip systems, which integrate microfluidics with organoid technology, provide an *in vitro* model capable of more faithfully recapitulating the dynamic tumor-immune interface. By co-culturing organoids and immune cells within these chips, researchers can investigate immune cell infiltration into tumor tissue, tumor cell killing mechanisms, and immune evasion strategies employed by malignant cells. Recent studies have further extended the application of this technology in precision cancer immunotherapy. For example, Park et al. (170) developed an Organoids-on-a-Chip platform that allows precise control of fluid shear stress, nutrient concentrations, and oxygen gradients, thereby providing a stable culture environment for lung cancer organoids (LCOs). This system more accurately mimics the physicochemical properties of the *in vivo* tumor microenvironment, offering a superior platform for studying immune cell migration and drug penetration. In addition, Poletti et al. (171) designed an innovative microfluidic device that supports the co-culture of LCOs with complex host microbial communities. This model not only simulates host-microbiome interactions but also elucidates how the microbiome modulates the local immune microenvironment to influence responses to cancer therapy, providing new insights for the development of microbiome-based immunotherapeutic strategies.

3D bioprinting enables the construction of tissues and organs with complex architectures through a layer-by-layer deposition process (84). Integrating 3D bioprinting with organoid technology facilitates the generation of vascularized organoids or immune cell co-culture constructs, thereby addressing the critical limitation of existing models—the lack of a functional vasculature. Vascularized organoids better recapitulate the nutrient supply and metabolic milieu of tumors, offering a more physiologically relevant *in vitro* model for drug discovery. Similarly, immune cell co-culture systems can mimic the cellular composition and spatial organization of immune populations within the tumor microenvironment, providing a powerful tool for immunotherapy research. Recent advances have substantially expanded the application of this integrated approach in building immunocompetent models. For instance, Heinrich et al. (172) employed extrusion-based bioprinting with a GelMA–gelatin bioink to fabricate a “mini-brain” model containing GL261 glioblastoma cells and RAW264.7 macrophages. This system successfully modeled paracrine and juxtacrine signaling between tumor cells and macrophages, driving macrophage migration toward tumor regions and inducing polarization. Compared with 2D cultures, key phenotypic changes were observed, including upregulation of Spp1 and loss of E-cadherin. In another study, Kim et al. (173) developed an innovative “bladder assembloid” model by bioprinting patient-derived tumor cells together with the four major stromal components of the bladder tumor microenvironment—cancer-associated fibroblasts (CAFs), endothelial cells, immune cells, and smooth muscle cells—thus reconstituting the intrinsic architecture of bladder cancer. This platform offers an exceptional tool for investigating interactions between tumor cells and diverse stromal cells, including immune populations, under *in vitro* conditions. Furthermore, Grunewald et al. (174) used projection-based bioprinting with GelMA to generate a neuroblastoma model. They observed that L1CAM-specific CAR-T cells infiltrated the 3D tumor model from the top to the bottom, displaying strong activation marked by increased interferon-gamma (IFNG) release and potent tumor-killing capacity.

## 6 Summary and outlook

Organoid technology acts as a crucial bridge between basic research and clinical translation, successfully simulating the native structure and microenvironment of tumors through three-dimensional culture systems. This approach demonstrates unique advantages in precision immunotherapy. Unlike traditional models, organoids retain tumor heterogeneity and intercellular interactions while integrating immune co-culture systems (such as T cells and macrophages). This high-fidelity reproduction of tumor-immune dynamics provides a reliable platform for studying mechanisms and evaluating the efficacy of therapies like immune checkpoint inhibitors and bispecific antibodies. Moreover, patient-derived organoid-based personalized drug sensitivity testing can accurately predict treatment responses, guiding the optimization of clinical strategies and significantly enhancing the precision and effectiveness of immunotherapy. These groundbreaking advancements lay a solid foundation for tumor biology research, the development of novel immunotherapies, and the practice of personalized medicine.

Looking ahead, tumor organoid technology is transitioning from a phase of technical validation to a critical stage of deep clinical integration and industrial application. Its developmental trajectory will no longer be confined to simple model construction but will increasingly focus on addressing clinical challenges. First, the core objective of next-generation organoid models will shift from “structural simulation” to “functional completeness.” While current models have made breakthroughs in reconstructing the immune microenvironment, several limitations continue to constrain their predictive accuracy. These include the long-term functional maintenance of T cells within three-dimensional matrices, precise control and stabilization of macrophage phenotypes, and the effective integration of vascular and lymphatic networks. Future breakthroughs will rely on more sophisticated bioengineering strategies. For instance, 3D bioprinting technologies could be employed to spatially arrange patient-derived endothelial cells, fibroblasts, and tumor cells to create perfusable “organoid-on-a-chip” systems. Alternatively, synthetic biology approaches could introduce reporter gene systems into organoids, enabling real-time, non-invasive monitoring of critical events such as T-cell activation and tumor cell killing. These endeavors aim to create a “patient avatar” that is not only morphologically similar but also functionally analogous to the original tumor. Such an advanced model would provide more predictive power for evaluating CAR-T therapies, immune checkpoint inhibitor combination regimens, and other emerging immunotherapeutic strategies. Secondly, deep data integration and intelligent interpretation will form the “intelligent core” of personalized prediction. Isolated drug sensitivity results from organoid assays alone represent merely discrete data points. The central challenge and opportunity within the future closed-loop “patient-organoid-multiomics-clinical decision” system lies in the multi-modal integration of organoid drug response data with real-time dynamic liquid biopsies (e.g., ctDNA), tumor neoantigen profiles, and immune cell states revealed by single-cell sequencing. This necessitates the development of a new generation of bioinformatics algorithms capable of extracting clinically actionable biomarkers from vast, heterogeneous datasets. For instance, machine learning models could be deployed to decipher why certain “cold tumors” that are unresponsive to PD-1 inhibitors in organoid models can be converted into “hot tumors” when combined with specific epigenetic modulators. Biomarkers derived from such organoid-based platforms will significantly optimize clinical trial enrollment criteria and refine treatment pathways. Finally, the clinical translation of this technology urgently needs to bridge the “valley of death” between validation and commercialization, which encompasses both ethical and commercial hurdles. Ethically, it is imperative to move beyond traditional one-time informed consent and establish dynamic consent frameworks adaptable to complex scenarios involving long-term organoid research, biobanking, and their use as disease models in drug development. Concurrently, the rich genomic information inherent in organoids demands stricter privacy protection and anonymization standards than conventional medical data, requiring clear delineation of data ownership and usage rights to prevent misuse in commercial applications. On the commercial front, the key challenge involves defining a clear regulatory approval pathway for organoids as companion diagnostics or *in vitro* clinical decision-support tools. This will require collaborative engagement with regulatory agencies to explore novel validation pathways potentially leveraging real-world data. Furthermore, rigorous health economics studies are essential to demonstrate cost-effectiveness—proving that the medical costs saved by avoiding ineffective treatments via organoid testing outweigh the assay costs—thus persuading healthcare payers. Additionally, clarifying intellectual property ownership (rights allocation among patients, hospitals, and technology developers) and innovating academia-industry collaboration models are foundational for sustainable translation. Only by constructing such a responsible innovation ecosystem can organoid technology truly transition from the laboratory to the bedside, fulfilling its promise to empower precision medicine and benefit patients.

## References

[1] Chiang IH Kuo HC Liao CT Kuo YC Yu SM Wang HM . An Ex vivo cultivation model for circulating tumor cells: The success rate and correlations with cancer response to therapy. BioMed J. (2025) 48:100819. doi: 10.1016/j.bj.2024.100819, PMID: 39622435 PMC11743102

[2] Yu S Zhang L Yang Y Wang M Liu T Ji W . Polydopamine-based resveratrol-hyaluronidase nanomedicine inhibited pancreatic cancer cell invasive phenotype in hyaluronic acid enrichment tumor sphere model. ACS Pharmacol Transl Sci. (2024) 7:1013–22. doi: 10.1021/acsptsci.3c00304, PMID: 38633596 PMC11020062

[3] Xu T Guo P He Y Pi C Wang Y Feng X . Application of curcumin and its derivatives in tumor multidrug resistance. Phytother Res. (2020) 34:2438–58. doi: 10.1002/ptr.6694, PMID: 32255545

[4] Shang Z Fan Y Xi S Zhang S Shen W Tao L . Arenobufagin enhances T-cell anti-tumor immunity in colorectal cancer by modulating HSP90β accessibility. Phytomedicine. (2024) 128:155497. doi: 10.1016/j.phymed.2024.155497, PMID: 38640855

[5] Lin DF Li HL Liu T Lv XF Xie CM Ou XM . Radiomic signatures associated with tumor immune heterogeneity predict survival in locally recurrent nasopharyngeal carcinoma. J Natl Cancer Inst. (2024) 116:1294–302. doi: 10.1093/jnci/djae081, PMID: 38637942

[6] Xu H Jiao D Liu A Wu K . Tumor organoids: applications in cancer modeling and potentials in precision medicine. J Hematol Oncol. (2022) 15:58. doi: 10.1186/s13045-022-01278-4, PMID: 35551634 PMC9103066

[7] Wang HM Zhang CY Peng KC Chen ZX Su JW Li YF . Using patient-derived organoids to predict locally advanced or metastatic lung cancer tumor response: A real-world study. Cell Rep Med. (2023) 4:100911. doi: 10.1016/j.xcrm.2022.100911, PMID: 36657446 PMC9975107

[8] Veninga V Voest EE . Tumor organoids: Opportunities and challenges to guide precision medicine. Cancer Cell. (2021) 39:1190–201. doi: 10.1016/j.ccell.2021.07.020, PMID: 34416168

[9] Neal JT Li X Zhu J Giangarra V Grzeskowiak CL Ju J . Organoid modeling of the tumor immune microenvironment. Cell. (2018) 175:1972–1988.e16. doi: 10.1016/j.cell.2018.11.021, PMID: 30550791 PMC6656687

[10] Dao V Yuki K Lo YH Nakano M Kuo CJ . Immune organoids: from tumor modeling to precision oncology. Trends Cancer. (2022) 8:870–80. doi: 10.1016/j.trecan.2022.06.001, PMID: 35773148 PMC9704769

[11] Liu Y Wu W Cai C Zhang H Shen H Han Y . Patient-derived xenograft models in cancer therapy: technologies and applications. Signal Transduct Target Ther. (2023) 8:160. doi: 10.1038/s41392-023-01419-2, PMID: 37045827 PMC10097874

[12] Abdolahi S Ghazvinian Z Muhammadnejad S Saleh M Asadzadeh Aghdaei H Baghaei K . Patient-derived xenograft (PDX) models, applications and challenges in cancer research. J Transl Med. (2022) 20:206. doi: 10.1186/s12967-022-03405-8, PMID: 35538576 PMC9088152

[13] Okada S Vaeteewoottacharn K Kariya R . Application of highly immunocompromised mice for the establishment of patient-derived xenograft (PDX) models. Cells. (2019) 8:889. doi: 10.3390/cells8080889, PMID: 31412684 PMC6721637

[14] Zhao Y Li S Zhu L Huang M Xie Y Song X . Personalized drug screening using patient-derived organoid and its clinical relevance in gastric cancer. Cell Rep Med. (2024) 5:101627. doi: 10.1016/j.xcrm.2024.101627, PMID: 38964315 PMC11293329

[15] Su ACY Ding X Lau HCH Kang X Li Q Wang X . *Lactococcus lactis HkyuLL* 10 suppresses colorectal tumourigenesis and restores gut microbiota through its generated alpha-mannosidase. Gut. (2024) 73:1478–88. doi: 10.1136/gutjnl-2023-330835, PMID: 38599786 PMC11347254

[16] Chaudhary N La Ferlita A Choudhary BS Jog E Kazi M Yahya S . Patient-derived organoids and xenografts uncover therapeutic vulnerabilities in colorectal signet ring cell carcinomas. Clin Cancer Res. (2025) 31:1359–73. doi: 10.1158/1078-0432.CCR-24-2329, PMID: 39879477

[17] Edmondson R Broglie JJ Adcock AF Yang L . Three-dimensional cell culture systems and their applications in drug discovery and cell-based biosensors. Assay Drug Dev Technol. (2014) 12:207–18. doi: 10.1089/adt.2014.573, PMID: 24831787 PMC4026212

[18] Yang Y Knight R Stephens P Zhang Y . Three-dimensional culture of oral progenitor cells: Effects on small extracellular vesicles production and proliferative function. J Oral Pathol Med. (2020) 49:342–9. doi: 10.1111/jop.12981, PMID: 31788854

[19] Sato T Vries RG Snippert HJ van de Wetering M Barker N Stange DE . Single Lgr5 stem cells build crypt-villus structures *in vitro* without a mesenchymal niche. Nature. (2009) 459:262–5. doi: 10.1038/nature07935, PMID: 19329995

[20] Prior N Inacio P Huch M . Liver organoids: from basic research to therapeutic applications. Gut. (2019) 68:2228–37. doi: 10.1136/gutjnl-2019-319256, PMID: 31300517 PMC6872443

[21] Corrò C Novellasdemunt L Li VSW . A brief history of organoids. Am J Physiol Cell Physiol. (2020) 319:C151–65. doi: 10.1152/ajpcell.00120.2020, PMID: 32459504 PMC7468890

[22] Carvalho MR Yan LP Li B Zhang CH He YL Reis RL . Gastrointestinal organs and organoids-on-a-chip: advances and translation into the clinics. Biofabrication. (2023) 15. doi: 10.1088/1758-5090/acf8fb, PMID: 37699408

[23] Polak R Zhang ET Kuo CJ . Cancer organoids 2.0: modelling the complexity of the tumour immune microenvironment. Nat Rev Cancer. (2024) 24:523–39. doi: 10.1038/s41568-024-00706-6, PMID: 38977835

[24] Bai L Zhou D Li G Liu J Chen X Su J . Engineering bone/cartilage organoids: strategy, progress, and application. Bone Res. (2024) 12:66. doi: 10.1038/s41413-024-00376-y, PMID: 39567500 PMC11579019

[25] Chen D Xu L Xuan M Chu Q Xue C . Unveiling the functional roles of patient-derived tumour organoids in assessing the tumour microenvironment and immunotherapy. Clin Transl Med. (2024) 14:e1802. doi: 10.1002/ctm2.1802, PMID: 39245957 PMC11381553

[26] Morizane R Lamers MM . Organoids in disease modeling and regenerative medicine. Cell Mol Life Sci. (2025) 82:169. doi: 10.1007/s00018-025-05692-y, PMID: 40257505 PMC12011692

[27] Powley IR Patel M Miles G Pringle H Howells L Thomas A . Patient-derived explants (PDEs) as a powerful preclinical platform for anti-cancer drug and biomarker discovery. Br J Cancer. (2020) 122:735–44. doi: 10.1038/s41416-019-0672-6, PMID: 31894140 PMC7078311

[28] Lv J Du X Wang M Su J Wei Y Xu C . Construction of tumor organoids and their application to cancer research and therapy. Theranostics. (2024) 14:1101–25. doi: 10.7150/thno.91362, PMID: 38250041 PMC10797287

[29] Sato K Zhang W Safarikia S Isidan A Chen AM Li P . Organoids and spheroids as models for studying cholestatic liver injury and cholangiocarcinoma. Hepatology. (2021) 74:491–502. doi: 10.1002/hep.31653, PMID: 33222247 PMC8529583

[30] Liu T Stephan T Chen P Keller-Findeisen J Chen J Riedel D . Multi-color live-cell STED nanoscopy of mitochondria with a gentle inner membrane stain. Proc Natl Acad Sci U S A. (2022) 119:e2215799119. doi: 10.1073/pnas.2215799119, PMID: 36534799 PMC9907107

[31] Qu J Kalyani FS Liu L Cheng T Chen L . Tumor organoids: synergistic applications, current challenges, and future prospects in cancer therapy. Cancer Commun (Lond). (2021) 41:1331–53. doi: 10.1002/cac2.12224, PMID: 34713636 PMC8696219

[32] Yoshida T Kates M Sopko NA Liu X Singh AK Bishai WR . Ex vivo culture of tumor cells from N-methyl-N-nitrosourea-induced bladder cancer in rats: Development of organoids and an immortalized cell line. Urol Oncol. (2018) 36:160.e23–160.e32. doi: 10.1016/j.urolonc.2017.11.024, PMID: 29288005

[33] Thorel L Perréard M Florent R Divoux J Coffy S Vincent A . Patient-derived tumor organoids: a new avenue for preclinical research and precision medicine in oncology. Exp Mol Med. (2024) 56:1531–51. doi: 10.1038/s12276-024-01272-5, PMID: 38945959 PMC11297165

[34] Kang MJ Ioannou S Lougheide Q Dittmar M Hsu Y Pastor-Soler NM . The study of intercalated cells using ex vivo techniques: primary cell culture, cell lines, kidney slices, and organoids. Am J Physiol Cell Physiol. (2024) 326:C229–51. doi: 10.1152/ajpcell.00479.2022, PMID: 37899748

[35] Ren F Wang L Wang Y Wang J Wang Y Song X . Single-cell transcriptome profiles the heterogeneity of tumor cells and microenvironments for different pathological endometrial cancer and identifies specific sensitive drugs. Cell Death Dis. (2024) 15:571. doi: 10.1038/s41419-024-06960-8, PMID: 39112478 PMC11306564

[36] Lee HS . Spatial and temporal tumor heterogeneity in gastric cancer: discordance of predictive biomarkers. J Gastric Cancer. (2025) 25:192–209. doi: 10.5230/jgc.2025.25.e3, PMID: 39822175 PMC11739643

[37] Huang J Wang X Ge S Lu X Sun C . Organoids as sophisticated tools for renal cancer research: extensive applications and promising prospects. Cell Mol Bioeng. (2024) 17:527–48. doi: 10.1007/s12195-024-00825-y, PMID: 39926385 PMC11799493

[38] Nicolini A Ferrari P . Involvement of tumor immune microenvironment metabolic reprogramming in colorectal cancer progression, immune escape, and response to immunotherapy. Front Immunol. (2024) 15:1353787. doi: 10.3389/fimmu.2024.1353787, PMID: 39119332 PMC11306065

[39] You L Wu Q . Cellular senescence in tumor immune escape: Mechanisms, implications, and therapeutic potential. Crit Rev Oncol Hematol. (2025) 208:104628. doi: 10.1016/j.critrevonc.2025.104628, PMID: 39864532

[40] Bergman PJ . Cancer immunotherapy. Vet Clin North Am Small Anim Pract. (2024) 54:441–68. doi: 10.1016/j.cvsm.2023.12.002, PMID: 38158304

[41] Liu W Cui Y Zheng X Yu K Sun G . Application status and future prospects of the PDX model in lung cancer. Front Oncol. (2023) 13:1098581. doi: 10.3389/fonc.2023.1098581, PMID: 37035154 PMC10080030

[42] Yoshida GJ . Applications of patient-derived tumor xenograft models and tumor organoids. J Hematol Oncol. (2020) 13:4. doi: 10.1186/s13045-019-0829-z, PMID: 31910904 PMC6947974

[43] Bleijs M van de Wetering M Clevers H Drost J . Xenograft and organoid model systems in cancerresearch. EMBO J. (2019) 38:e101654. doi: 10.15252/embj.2019101654, PMID: 31282586 PMC6670015

[44] Jin X Demere Z Nair K Ali A Ferraro GB Natoli T . A metastasis map of human cancer cell lines. Nature. (2020) 588:331–6. doi: 10.1038/s41586-020-2969-2, PMID: 33299191 PMC8439149

[45] Gunti S Hoke ATK Vu KP London NR Jr . Organoid and spheroid tumor models: techniques and applications. Cancers (Basel). (2021) 13:874. doi: 10.3390/cancers13040874, PMID: 33669619 PMC7922036

[46] Ren X Chen W Yang Q Li X Xu L . Patient-derived cancer organoids for drug screening: Basic technology and clinical application. J Gastroenterol Hepatol. (2022) 37:1446–54. doi: 10.1111/jgh.15930, PMID: 35771719

[47] Liu J Huang X Huang L Huang J Liang D Liao L . Organoid: next-generation modeling of cancer research and drug development. Front Oncol. (2022) 11:826613. doi: 10.3389/fonc.2021.826613, PMID: 35155215 PMC8831330

[48] Jeong SR Kang M . Exploring tumor-immune interactions in co-culture models of T cells and tumor organoids derived from patients. Int J Mol Sci. (2023) 24:14609. doi: 10.3390/ijms241914609, PMID: 37834057 PMC10572813

[49] Nie X Liang Z Li K Yu H Huang Y Ye L . Novel organoid model in drug screening: Past, present, and future. Liver Res. (2021) 5:72–8. doi: 10.1016/j.livres.2021.05.003, PMID: 39959346 PMC11791835

[50] Yang Y Cui J Kong Y Hou Y Ma C . Organoids: new frontiers in tumor immune microenvironment research. Front Immunol. (2024) 15:1422031. doi: 10.3389/fimmu.2024.1422031, PMID: 39136020 PMC11317300

[51] Cao R Li NT Latour S Cadavid JL Tan CM Forman A . An automation workflow for high-throughput manufacturing and analysis of scaffold-supported 3D tissue arrays. Adv Healthc Mater. (2023) 12:e2202422. doi: 10.1002/adhm.202202422, PMID: 37086259 PMC11468893

[52] Zhou B Feng Z Xu J Xie J . Organoids: approaches and utility in cancer research. Chin Med J (Engl). (2023) 136:1783–93. doi: 10.1097/CM9.0000000000002477, PMID: 37365679 PMC10406116

[53] Booij TH Cattaneo CM Hirt CK . Tumor organoids as a research tool: how to exploit them. Cells. (2022) 11:3440. doi: 10.3390/cells11213440, PMID: 36359838 PMC9653788

[54] Yang L Yang S Li X Li B Li Y Zhang X . Tumor organoids: From inception to future in cancer research. Cancer Lett. (2019) 454:120–33. doi: 10.1016/j.canlet.2019.04.005, PMID: 30981763

[55] Kim SY van de Wetering M Clevers H Sanders K . The future of tumor organoids in precision therapy. Trends Cancer. (2025) 11:665–75. doi: 10.1016/j.trecan.2025.03.005, PMID: 40185656

[56] Urbano PCM Angus HCK Gadeock S Schultz M Kemp RA . Assessment of source material for human intestinal organoid culture for research and clinical use. BMC Res Notes. (2022) 15:35. doi: 10.1186/s13104-022-05925-4, PMID: 35144661 PMC8830126

[57] Broutier L Mastrogiovanni G Verstegen MM Francies HE Gavarró LM Bradshaw CR . Human primary liver cancer-derived organoid cultures for disease modeling and drug screening. Nat Med. (2017) 23:1424–35. doi: 10.1038/nm.4438, PMID: 29131160 PMC5722201

[58] Mo S Tang P Luo W Zhang L Li Y Hu X . Patient-derived organoids from colorectal cancer with paired liver metastasis reveal tumor heterogeneity and predict response to chemotherapy. Adv Sci (Weinh). (2022) 9:e2204097. doi: 10.1002/advs.202204097, PMID: 36058001 PMC9631073

[59] Wang L Yu Y Fang Y Li Y Yu W Wang Z . Malignant pleural effusion facilitates the establishment and maintenance of tumor organoid biobank with multiple patient-derived lung tumor cell sources. Exp Hematol Oncol. (2024) 13:115. doi: 10.1186/s40164-024-00581-9, PMID: 39548571 PMC11566167

[60] Lin CN Liang YL Tsai HF Wu PY Huang LY Lin YH . Adipocyte pyroptosis occurs in omental tumor microenvironment and is associated with chemoresistance of ovarian cancer. J BioMed Sci. (2024) 31:62. doi: 10.1186/s12929-024-01051-4, PMID: 38862973 PMC11167873

[61] Sailer V Pauli C Merzier EC Mosquera JM Beltran H Rubin MA . On-site cytology for development of patient-derived three-dimensional organoid cultures - A pilot study. Anticancer Res. (2017) 37:1569–73. doi: 10.21873/anticanres.11486, PMID: 28373416

[62] Pleguezuelos-Manzano C Puschhof J van den Brink S Geurts V Beumer J Clevers H . Establishment and culture of human intestinal organoids derived from adult stem cells. Curr Protoc Immunol. (2020) 130:e106. doi: 10.1002/cpim.106, PMID: 32940424 PMC9285512

[63] Sato T Stange DE Ferrante M Vries RG Van Es JH Van den Brink S . Long-term expansion of epithelial organoids from human colon, adenoma, adenocarcinoma, and Barrett’s epithelium. Gastroenterology. (2011) 141:1762–72. doi: 10.1053/j.gastro.2011.07.050, PMID: 21889923

[64] Cordts SC Yuki K Henao Echeverri MF Narasimhan B Kuo CJ Tang SKY . Microdissection tools to generate organoids for modeling the tumor immune microenvironment. Microsystems nanoengineering. (2024) 10:126. doi: 10.1038/s41378-024-00756-8, PMID: 39251611 PMC11385579

[65] Boilève A Cartry J Goudarzi N Bedja S Mathieu JRR Bani MA . Organoids for functional precision medicine in advanced pancreatic cancer. Gastroenterology. (2024) 167:961–976.e13. doi: 10.1053/j.gastro.2024.05.032, PMID: 38866343

[66] Vlachogiannis G Hedayat S Vatsiou A Jamin Y Fernández-Mateos J Khan K . Patient-derived organoids model treatment response of metastatic gastrointestinal cancers. Science. (2018) 359:920–6. doi: 10.1126/science.aao2774, PMID: 29472484 PMC6112415

[67] Estridge RC O’Neill JE Keung AJ . Matrigel tunes H9 stem cell-derived human cerebral organoid development. Organoids. (2023) 2:165–76. doi: 10.3390/organoids2040013, PMID: 38196836 PMC10776236

[68] Ito F Kato K Yanatori I Maeda Y Murohara T Toyokuni S . Matrigel-based organoid culture of Malignant mesothelioma reproduces cisplatin sensitivity through CTR1. BMC Cancer. (2023) 23:487. doi: 10.1186/s12885-023-10966-4, PMID: 37254056 PMC10230733

[69] Ma L Li J Nie Q Zhang Q Liu S Ge D . Organoid culture of human prostate cancer cell lines LNCaP and C4-2B. Am J Clin Exp Urol. (2017) 5:25–33., PMID: 29181435 PMC5698596

[70] Kozlowski MT Crook CJ Ku HT . Towards organoid culture without Matrigel. Commun Biol. (2021) 4:1387. doi: 10.1038/s42003-021-02910-8, PMID: 34893703 PMC8664924

[71] Luo X Fong ELS Zhu C Lin QXX Xiong M Li A . Hydrogel-based colorectal cancer organoid co-culture models. Acta Biomater. (2021) 132:461–472. doi: 10.1016/j.actbio.2020.12.037, PMID: 33388439

[72] Yi SA Zhang Y Rathnam C Pongkulapa T Lee KB . Bioengineering approaches for the advanced organoid research. Adv Mater. (2021) 33:e2007949. doi: 10.1002/adma.202007949, PMID: 34561899 PMC8682947

[73] Yu T Yang Q Peng B Gu Z Zhu D . Vascularized organoid-on-a-chip: design, imaging, and analysis. Angiogenesis. (2024) 27:147–72. doi: 10.1007/s10456-024-09905-z, PMID: 38409567

[74] Jung SY You HJ Kim MJ Ko G Lee S Kang KS . Wnt-activating human skin organoid model of atopic dermatitis induced by *Staphylococcus aureus* and its protective effects by Cutibacterium acnes. iScience. (2022) 25:105150. doi: 10.1016/j.isci.2022.105150, PMID: 36193049 PMC9526179

[75] Castaneda DC Jangra S Yurieva M Martinek J Callender M Coxe M . Protocol for establishing primary human lung organoid-derived air-liquid interface cultures from cryopreserved human lung tissue. STAR Protoc. (2023) 4:102735. doi: 10.1016/j.xpro.2023.102735, PMID: 37991921 PMC10696416

[76] Wang Z Yu T Hou Y Zhou W Ding Y Nie H . Mesenchymal stem cell therapy for ALI/ARDS: therapeutic potential and challenges. Curr Pharm Des. (2022) 28:2234–40. doi: 10.2174/1381612828666220707104356, PMID: 35796453

[77] Doyle AD Masuda MY Pyon GC Luo H Putikova A LeSuer WE . Detergent exposure induces epithelial barrier dysfunction and eosinophilic inflammation in the esophagus. Allergy. (2023) 78:192–201. doi: 10.1111/all.15457, PMID: 35899466 PMC9797443

[78] Hu Y Zhang H Wang S Cao L Zhou F Jing Y . Bone/cartilage organoid on-chip: Construction strategy and application. Bioact Mater. (2023) 25:29–41. doi: 10.1016/j.bioactmat.2023.01.016, PMID: 37056252 PMC10087111

[79] Kroll KT Homan KA Uzel SGM Mata MM Wolf KJ Rubins JE . A perfusable, vascularized kidney organoid-on-chip model. Biofabrication. (2024) 16. doi: 10.1088/1758-5090/ad5ac0, PMID: 38906132

[80] Xiang T Wang J Li H . Current applications of intestinal organoids: a review. Stem Cell Res Ther. (2024) 15:155. doi: 10.1186/s13287-024-03768-3, PMID: 38816841 PMC11140936

[81] Shukla P Yeleswarapu S Heinrich MA Prakash J Pati F . Mimicking tumor microenvironment by 3D bioprinting: 3D cancer modeling. Biofabrication. (2022) 14. doi: 10.1088/1758-5090/ac6d11, PMID: 35512666

[82] Zhang YS Arneri A Bersini S Shin SR Zhu K Goli-Malekabadi Z . Bioprinting 3D microfibrous scaffolds for engineering endothelialized myocardium and heart-on-a-chip. Biomaterials. (2016) 110:45–59. doi: 10.1016/j.biomaterials.2016.09.003, PMID: 27710832 PMC5198581

[83] Bertsch P Diba M Mooney DJ Leeuwenburgh SCG . Self-healing injectable hydrogels for tissue regeneration. Chem Rev. (2023) 123:834–73. doi: 10.1021/acs.chemrev.2c00179, PMID: 35930422 PMC9881015

[84] Zhang Z Chen X Gao S Fang X Ren S . 3D bioprinted tumor model: a prompt and convenient platform for overcoming immunotherapy resistance by recapitulating the tumor microenvironment. Cell Oncol. (2024) 47:1113–26. doi: 10.1007/s13402-024-00935-9, PMID: 38520648 PMC11322267

[85] Shan H Chen M Zhao S Wei X Zheng M Li Y . Acoustic virtual 3D scaffold for direct-interacting tumor organoid-immune cell coculture systems. Sci Adv. (2024) 10:eadr4831. doi: 10.1126/sciadv.adr4831, PMID: 39576870 PMC11584020

[86] Santos AJM van Unen V Lin Z Chirieleison SM Ha N Batish A . A human autoimmune organoid model reveals IL-7 function in coeliac disease. Nature. (2024) 632:401–10. doi: 10.1038/s41586-024-07716-2, PMID: 39048815 PMC11747932

[87] Nickl V Eck J Goedert N Hübner J Nerreter T Hagemann C . Characterization and optimization of the tumor microenvironment in patient-derived organotypic slices and organoid models of glioblastoma. Cancers (Basel). (2023) 15:2698. doi: 10.3390/cancers15102698, PMID: 37345035 PMC10216617

[88] Öhlund D Handly-Santana A Biffi G Elyada E Almeida AS Ponz-Sarvise M . Distinct populations of inflammatory fibroblasts and myofibroblasts in pancreatic cancer. J Exp Med. (2017) 214:579–96. doi: 10.1084/jem.20162024, PMID: 28232471 PMC5339682

[89] Yang Q Li M Yang X Zhang Y Wang J Chen L . Flourishing tumor organoids: History, emerging technology, and application. Bioengineering Trans Med. (2023) 8:e10559. doi: 10.1002/btm2.10559, PMID: 37693042 PMC10487342

[90] Nguyen NTB Gevers S Kok RNU Burgering LM Neikes H Akkerman N . Lactate controls cancer stemness and plasticity through epigenetic regulation. Cell Metab. (2025) 37:903–919.e10. doi: 10.1016/j.cmet.2025.01.002, PMID: 39933514

[91] Bues J Biočanin M Pezoldt J Dainese R Chrisnandy A Rezakhani S . Deterministic scRNA-seq captures variation in intestinal crypt and organoid composition. Nat Methods. (2022) 19:323–30. doi: 10.1038/s41592-021-01391-1, PMID: 35165449

[92] Zhu L Fan Y Huang X Chen T Xu X Xu F . Patent bibliometric analysis for global trend of organoid technologies in the past decade. iScience. (2022) 25:104728. doi: 10.1016/j.isci.2022.104728, PMID: 35880045 PMC9307668

[93] Ye W Luo C Li C Huang J Liu F . Organoids to study immune functions, immunological diseases and immunotherapy. Cancer Lett. (2020) 477:31–40. doi: 10.1016/j.canlet.2020.02.027, PMID: 32112908

[94] Li G Ghosh S Park J Shin H Garige M Reaman G . A mouse pancreatic organoid model to compare PD-L1 blocking antibodies. MAbs. (2022) 14:2139886. doi: 10.1080/19420862.2022.2139886, PMID: 36334035 PMC9639566

[95] Gao M Lin M Moffitt RA Salazar MA Park J Vacirca J . Direct therapeutic targeting of immune checkpoint PD-1 in pancreatic cancer. Br J Cancer. (2019) 120:88–96. doi: 10.1038/s41416-018-0298-0, PMID: 30377341 PMC6325157

[96] Votanopoulos K Forsythe S Sivakumar H Mazzocchi A Aleman J Miller L . Model of patient-specific immune-enhanced organoids for immunotherapy screening: feasibility study. Ann Surg Oncol. (2019) 27:1956–67. doi: 10.1245/s10434-019-08143-8, PMID: 31858299 PMC7474462

[97] Kastenschmidt JM Sureshchandra S Jain A Hernandez-Davies JE de Assis R Wagoner ZW . Influenza vaccine format mediates distinct cellular and antibody responses in human immune organoids. Immunity. (2023) 56:1910–1926.e7. doi: 10.1016/j.immuni.2023.06.019, PMID: 37478854 PMC10433940

[98] Wagar LE Salahudeen A Constantz CM Wendel BS Lyons MM Mallajosyula V . Modeling human adaptive immune responses with tonsil organoids. Nat Med. (2021) 27:125–35. doi: 10.1038/s41591-020-01145-0, PMID: 33432170 PMC7891554

[99] Ren Y Manoharan T Liu B Cheng CZM En Siew B Cheong WK . Circular RNA as a source of neoantigens for cancer vaccines. J Immunother Cancer. (2024) 12:e008402. doi: 10.1136/jitc-2023-008402, PMID: 38508656 PMC10952939

[100] Forsythe SD Erali RA Sasikumar S Laney P Shelkey E D'Agostino R Jr . Organoid platform in preclinical investigation of personalized immunotherapy efficacy in appendiceal cancer: feasibility study. Clin Cancer Res. (2021) 27:5141–50. doi: 10.1158/1078-0432.CCR-21-0982, PMID: 34210684 PMC8720262

[101] Valančiūtė A Mathieson L O’Connor RA Scott JI Vendrell M Dorward DA . Phototherapeutic induction of immunogenic cell death and CD8+ T cell-granzyme B mediated cytolysis in human lung cancer cells and organoids. Cancers (Basel). (2022) 14:4119. doi: 10.3390/cancers14174119, PMID: 36077656 PMC9454585

[102] Lee SH Hu W Matulay JT Silva MV Owczarek TB Kim K . Tumor evolution and drug response in patient-derived organoid models of bladder cancer. Cell. (2018) 173:515–528.e17. doi: 10.1016/j.cell.2018.03.017, PMID: 29625057 PMC5890941

[103] Al Shihabi A Tebon PJ Nguyen HTL Chantharasamee J Sartini S Davarifar A . The landscape of drug sensitivity and resistance in sarcoma. Cell Stem Cell. (2024) 31:1524–1542.e4. doi: 10.1016/j.stem.2024.08.010, PMID: 39305899 PMC12318355

[104] Xu R Zhou X Wang S Trinkle C . Tumor organoid models in precision medicine and investigating cancer-stromal interactions. Pharmacol Ther. (2021) 218:107668. doi: 10.1016/j.pharmthera.2020.107668, PMID: 32853629 PMC7855432

[105] Kastenschmidt JM Schroers-Martin JG Sworder BJ Sureshchandra S Khodadoust MS Liu CL . A human lymphoma organoid model for evaluating and targeting the follicular lymphoma tumor immune microenvironment. Cell Stem Cell. (2024) 31:410–420.e4. doi: 10.1016/j.stem.2024.01.012, PMID: 38402619 PMC10960522

[106] Hu LF Yang X Lan HR Fang XL Chen XY Jin KT . Preclinical tumor organoid models in personalized cancer therapy: Not everyone fits the mold. Exp Cell Res. (2021) 408:112858. doi: 10.1016/j.yexcr.2021.112858, PMID: 34600901

[107] Ren YF Ma Q Zeng X Huang CX Ren JL Li F . Single-cell RNA sequencing reveals immune microenvironment niche transitions during the invasive and metastatic processes of ground-glass nodules and part-solid nodules in lung adenocarcinoma. Mol Cancer. (2024) 23:263. doi: 10.1186/s12943-024-02177-7, PMID: 39580469 PMC11585206

[108] Yu Y Liang Y Xie F Zhang Z Zhang P Zhao X . Tumor-associated macrophage enhances PD-L1-mediated immune escape of bladder cancer through PKM2 dimer-STAT3 complex nuclear translocation. Cancer Lett. (2024) 593:216964. doi: 10.1016/j.canlet.2024.216964, PMID: 38762193

[109] Ho T Msallam R . Tissues and tumor microenvironment (TME) in 3D: models to shed light on immunosuppression in cancer. Cells. (2021) 10:831. doi: 10.3390/cells10040831, PMID: 33917037 PMC8067689

[110] Li X Fu G Zhang L Guan R Tang P Zhang J . Assay establishment and validation of a high-throughput organoid-based drug screening platform. Stem Cell Res Ther. (2022) 13:219. doi: 10.1186/s13287-022-02902-3, PMID: 35619149 PMC9137096

[111] Pinezich MR O’Neill JD Guenthart BA Kim J Vila OF Ma SP . Theranostic methodology for ex vivo donor lung rehabilitation. Med. (2025) 6:100644. doi: 10.1016/j.medj.2025.100644, PMID: 40154476

[112] Branciforti F Salvi M D’Agostino F Marzola F Cornacchia S De Titta MO . Segmentation and multi-timepoint tracking of 3D cancer organoids from optical coherence tomography images using deep neural networks. Diagnostics (Basel). (2024) 14:1217. doi: 10.3390/diagnostics14121217, PMID: 38928633 PMC11203156

[113] Zhang CJ Meyer SR O’Meara MJ Huang S Capeling MM Ferrer-Torres D . A human liver organoid screening platform for DILI risk prediction. J Hepatol. (2023) 78:998–1006. doi: 10.1016/j.jhep.2023.01.019, PMID: 36738840 PMC11268729

[114] Ji S Feng L Fu Z Wu G Wu Y Lin Y . Pharmaco-proteogenomic characterization of liver cancer organoids for precision oncology. Sci Transl Med. (2023) 15:eadg3358. doi: 10.1126/scitranslmed.adg3358, PMID: 37494474 PMC10949980

[115] Xu H Lyu X Yi M Zhao W Song Y Wu K . Organoid technology and applications in cancer research. J Hematol Oncol. (2018) 11:116. doi: 10.1186/s13045-018-0662-9, PMID: 30219074 PMC6139148

[116] Wang J Li X Chen H . Organoid models in lung regeneration and cancer. Cancer Lett. (2020) 475:129–35. doi: 10.1016/j.canlet.2020.01.030, PMID: 32032677

[117] Kye Y Nagineni L Gadad S Ramirez F Riva H Fernandez L . The identification and clinical applications of mutated antigens in the era of immunotherapy. Cancers. (2022) 14:4255. doi: 10.3390/cancers14174255, PMID: 36077792 PMC9454936

[118] Westcott PMK Sacks NJ Schenkel JM Ely ZA Smith O Hauck H . Low neoantigen expression and poor T-cell priming underlie early immune escape in colorectal cancer. Nat Cancer. (2021) 2:1071–85. doi: 10.1038/s43018-021-00247-z, PMID: 34738089 PMC8562866

[119] Vazquez-Armendariz AI Tata PR . Recent advances in lung organoid development and applications in disease modeling. J Clin Invest. (2023) 133:e170500. doi: 10.1172/JCI170500, PMID: 37966116 PMC10645385

[120] Ji DB Wu AW . Organoid in colorectal cancer: progress and challenges. Chin Med J (Engl). (2020) 133:1971–7. doi: 10.1097/CM9.0000000000000882, PMID: 32826461 PMC7462208

[121] Mao Y Wang W Yang J Zhou X Lu Y Gao J . Drug repurposing screening and mechanism analysis based on human colorectal cancer organoids. Protein Cell. (2024) 15:285–304. doi: 10.1093/procel/pwad038, PMID: 37345888 PMC10984622

[122] Li J Liu J Xia W Yang H Sha W Chen H . Deciphering the tumor microenvironment of colorectal cancer and guiding clinical treatment with patient-derived organoid technology: progress and challenges. Technol Cancer Res Treat. (2024) 23:15330338231221856. doi: 10.1177/15330338231221856, PMID: 38225190 PMC10793199

[123] Cristobal A van den Toorn HWP van de Wetering M Clevers H Heck AJR Mohammed S . Personalized proteome profiles of healthy and tumor human colon organoids reveal both individual diversity and basic features of colorectal cancer. Cell Rep. (2017) 18:263–74. doi: 10.1016/j.celrep.2016.12.016, PMID: 28052255

[124] Zheng X Li J Sheng J Zhuo Q Du Q Xu Y . Exploration of organoid in breast cancer related research. Chin J Biotechnol. (2021) 37:395–403. doi: 10.13345/j.cjb.200285, PMID: 33645143

[125] Liu Y Hu Y Xue J Li J Yi J Bu J . Advances in immunotherapy for triple-negative breast cancer. Mol Cancer. (2023) 22:145. doi: 10.1186/s12943-023-01850-7, PMID: 37660039 PMC10474743

[126] Guan D Liu X Shi Q He B Zheng C Meng X . Breast cancer organoids and their applications for precision cancer immunotherapy. World J Surg Oncol. (2023) 21:343. doi: 10.1186/s12957-023-03231-2, PMID: 37884976 PMC10601270

[127] Huang C Jin H . Progress and perspective of organoid technology in breast cancer research. Chin Med J. (2024) 137:2157–68. doi: 10.1097/CM9.0000000000002889, PMID: 38185826 PMC11407818

[128] Zhang Q Wang M You L Chen C Feng J Song M . Research progress and application status of organoid in breast cancer subtypes. Biomolecules Biomedicine. (2025) 25:976–85. doi: 10.17305/bb.2024.11450, PMID: 39720912 PMC11984363

[129] Önder CE Moustafa-Oglou M Schröder SM Hartkopf AD Koch A Seitz CM . Precision immunotherapy utilizing adapter CAR-T cells (AdCAR-T) in metastatic breast cancer leads to target specific lysis. Cancers. (2023) 16:168. doi: 10.3390/cancers16010168, PMID: 38201595 PMC10778501

[130] Yokota E Iwai M Yukawa T Yoshida M Naomoto Y Haisa M . Clinical application of a lung cancer organoid (tumoroid) culture system. NPJ Precis Oncol. (2021) 5:29. doi: 10.1038/s41698-021-00166-3, PMID: 33846488 PMC8042017

[131] Tian H Ren J Mou R Jia Y . Application of organoids in precision immunotherapy of lung cancer (Review). Oncol Lett. (2023) 26:484. doi: 10.3892/ol.2023.14071, PMID: 37818130 PMC10561155

[132] Huang Z Li B Wang Y Xue J Wei Z Liang N . Application and research progress of lung cancer organoid in precision medicine for lung cancer. Chin J Lung Cancer. (2024) 27:276–82. doi: 10.3779/j.issn.1009-3419.2024.106.07, PMID: 38769830 PMC11110296

[133] Lee E Lee SY Seong YJ Ku B Cho HJ Kim K . Lung cancer organoid-based drug evaluation models and new drug development application trends. Trans Lung Cancer Res. (2024) 13:3741–63. doi: 10.21037/tlcr-24-603, PMID: 39830742 PMC11736608

[134] Kumar V Ramnarayanan K Sundar R Padmanabhan N Srivastava S Koiwa M . Single-cell atlas of lineage states, tumor microenvironment, and subtype-specific expression programs in gastric cancer. Cancer Discov. (2022) 12:670–91. doi: 10.1158/2159-8290.CD-21-0683, PMID: 34642171 PMC9394383

[135] Lo YH Kolahi KS Du Y Chang CY Krokhotin A Nair A . A CRISPR/cas9-engineered *ARID1A*-deficient human gastric cancer organoid model reveals essential and nonessential modes of oncogenic transformation. Cancer Discov. (2021) 11:1562–81. doi: 10.1158/2159-8290.CD-20-1109, PMID: 33451982 PMC8346515

[136] Huo C Zhang X Gu Y Wang D Zhang S Liu T . Organoids: construction and application in gastric cancer. Biomolecules. (2023) 13:875. doi: 10.3390/biom13050875, PMID: 37238742 PMC10216466

[137] Wang Q Guo F Zhang Q Hu T Jin Y Yang Y . Organoids in gastrointestinal diseases: from bench to clinic. MedComm. (2024) 5:e574. doi: 10.1002/mco2.574, PMID: 38948115 PMC11214594

[138] Fan S Yin J Wang M Guan W . Establishment and application of gastric and gastric cancer organoids. Chin J Gastrointestinal Surg. (2018) 21:1315–20., PMID: 30506544

[139] Miao X Wang C Chai C Tang H Hu J Zhao Z . Establishment of gastric cancer organoid and its application in individualized therapy. Oncol Lett. (2022) 24:447. doi: 10.3892/ol.2022.13567, PMID: 36420067 PMC9647790

[140] Ren X Chen W Yang Q Li X Xu L . Patient-derived cancer organoids for drug screening: Basic technology and clinical application. J Gastroenterol Hepatol. (2022) 37:1446–54. doi: 10.1111/jgh.15930, PMID: 35771719

[141] Meister MT Groot Koerkamp MJA de Souza T Breunis WB Frazer-Mendelewska E Brok M . Mesenchymal tumor organoid models recapitulate rhabdomyosarcoma subtypes. EMBO Mol Med. (2022) 14:e16001. doi: 10.15252/emmm.202216001, PMID: 35916583 PMC9549731

[142] Yu YY Zhu YJ Xiao ZZ Chen YD Chang XS Liu YH . The pivotal application of patient-derived organoid biobanks for personalized treatment of gastrointestinal cancers. biomark Res. (2022) 10:73. doi: 10.1186/s40364-022-00421-0, PMID: 36207749 PMC9547471

[143] Goncalves B Liu S Zhang X Fan A Ou L Xu X . Unveiling therapeutic opportunities with melanoma patient-derived organoid models. J Visualized Experiments. (2024) 211):10.3791/66509. doi: 10.3791/66509, PMID: 39311587 PMC11539850

[144] Zhou LF Liao HY Han Y Zhao Y . The use of organoids in creating immune microenvironments and treating gynecological tumors. J Trans Med. (2024) 22:856. doi: 10.1186/s12967-024-05649-y, PMID: 39313812 PMC11421176

[145] Xu J Pham MD Corbo V Ponz-Sarvise M Oni T Öhlund D . Advancing pancreatic cancer research and therapeutics: the transformative role of organoid technology. Exp Mol Med. (2025) 57:50–8. doi: 10.1038/s12276-024-01378-w, PMID: 39814914 PMC11799150

[146] Yang H Cheng J Zhuang H Xu H Wang Y Zhang T . Pharmacogenomic profiling of intra-tumor heterogeneity using a large organoid biobank of liver cancer. Cancer Cell. (2024) 42:535–551.e8. doi: 10.1016/j.ccell.2024.03.004, PMID: 38593780

[147] Sun L Kang X Ju H Wang C Yang G Wang R . A human mucosal melanoma organoid platform for modeling tumor heterogeneity and exploring immunotherapy combination options. Sci Adv. (2023) 9:eadg6686. doi: 10.1126/sciadv.adg6686, PMID: 37889972 PMC10610903

[148] Senkowski W Gall-Mas L Falco MM Li Y Lavikka K Kriegbaum MC . A platform for efficient establishment and drug-response profiling of high-grade serous ovarian cancer organoids. Dev Cell. (2023) 58:1106–1121.e7. doi: 10.1016/j.devcel.2023.04.012, PMID: 37148882 PMC10281085

[149] Li Y Liao W Sun L . Application of tumor organoids simulating the tumor microenvironment in basic and clinical research of tumor immunotherapy. J Cent South University. Med Sci. (2024) 49:1316–26. doi: 10.11817/j.issn.1672-7347.2024.240187, PMID: 39788520 PMC11628225

[150] Yao N Jing N Lin J Niu W Yan W Yuan H . Patient-derived tumor organoids for cancer immunotherapy: culture techniques and clinical application. Investigational New Drugs. (2025) 43:394–404. doi: 10.1007/s10637-025-01523-w, PMID: 40232355 PMC12048417

[151] Wu P Han J Gong Y Liu C Yu H Xie N . Nanoparticle-based drug delivery systems targeting tumor microenvironment for cancer immunotherapy resistance: current advances and applications. Pharmaceutics. (2022) 14:1990. doi: 10.3390/pharmaceutics14101990, PMID: 36297426 PMC9612242

[152] Wu Y Verma V Gay CM Chen Y Liang F Lin Q . Neoadjuvant immunotherapy for advanced, resectable non-small cell lung cancer: A systematic review and meta-analysis. Cancer. (2023) 129:1969–85. doi: 10.1002/cncr.34755, PMID: 36994945

[153] Cortes J Haiderali A Huang M Pan W Schmid P Akers KG . Neoadjuvant immunotherapy and chemotherapy regimens for the treatment of high-risk, early-stage triple-negative breast cancer: a systematic review and network meta-analysis. BMC Cancer. (2023) 23:792. doi: 10.1186/s12885-023-11293-4, PMID: 37612624 PMC10463750

[154] Xu H Jiao D Liu A Wu K . Tumor organoids: applications in cancer modeling and potentials in precision medicine. J Hematol Oncol. (2022) 15:58. doi: 10.1186/s13045-022-01278-4, PMID: 35551634 PMC9103066

[155] Qu S Xu R Yi G Li Z Zhang H Qi S . Patient-derived organoids in human cancer: a platform for fundamental research and precision medicine. Mol Biomedicine. (2024) 5:6. doi: 10.1186/s43556-023-00165-9, PMID: 38342791 PMC10859360

[156] Skardal A Aleman J Forsythe S Rajan S Murphy S Devarasetty M . Drug compound screening in single and integrated multi-organoid body-on-a-chip systems. Biofabrication. (2020) 12:025017. doi: 10.1088/1758-5090/ab6d36, PMID: 32101533

[157] Liu HD Xia BR Jin MZ Lou G . Organoid of ovarian cancer: genomic analysis and drug screening. Clin Transl Oncol. (2020) 22:1240–51. doi: 10.1007/s12094-019-02276-8, PMID: 31939100 PMC7316695

[158] Boretto M Maenhoudt N Luo X Hennes A Boeckx B Bui B . Patient-derived organoids from endometrial disease capture clinical heterogeneity and are amenable to drug screening. Nat Cell Biol. (2019) 21:1041–51. doi: 10.1038/s41556-019-0360-z, PMID: 31371824

[159] Xie X Li X Song W . Tumor organoid biobank-new platform for medical research. Sci Rep. (2023) 13:1819. doi: 10.1038/s41598-023-29065-2, PMID: 36725963 PMC9892604

[160] Dalvin LA Andrews-Pfannkoch CM Miley DR Hogenson TL Erickson SA Malpotra S . Novel uveal melanoma patient-derived organoid models recapitulate human disease to support translational research. Invest Ophthalmol Vis Sci. (2024) 65:60. doi: 10.1167/iovs.65.13.60, PMID: 39601636 PMC11605663

[161] Pang L Huang Y Zhuang W Zhang Y Liao J Hao Y . Co-occurring EGFR p.E709X Mutation Mediates Primary Resistance to the Third-Generation EGFR-TKIs in EGFR p.G719X-Mutant Patients with Advanced NSCLC. Clin Cancer Res. (2024) 30:2636–46. doi: 10.1158/1078-0432.CCR-23-3302, PMID: 38578683

[162] Yao Y Xu X Yang L Zhu J Wan J Shen L . Patient-derived organoids predict chemoradiation responses of locally advanced rectal cancer. Cell Stem Cell. (2020) 26:17–26.e6. doi: 10.1016/j.stem.2019.10.010, PMID: 31761724

[163] Wang Y Jiang T Qin Z Jiang J Wang Q Yang S . HER2 exon 20 insertions in non-small-cell lung cancer are sensitive to the irreversible pan-HER receptor tyrosine kinase inhibitor pyrotinib. Ann Oncol. (2019) 30:447–55. doi: 10.1093/annonc/mdy542, PMID: 30596880 PMC7360147

[164] Ooft SN Weeber F Dijkstra KK McLean CM Kaing S van Werkhoven E . Patient-derived organoids can predict response to chemotherapy in metastatic colorectal cancer patients. Sci Transl Med. (2019) 11:eaay2574. doi: 10.1126/scitranslmed.aay2574, PMID: 31597751

[165] Driehuis E van Hoeck A Moore K Kolders S Francies HE Gulersonmez MC . Pancreatic cancer organoids recapitulate disease and allow personalized drug screening. Proc Natl Acad Sci U S A. (2019) 116:26580–90. doi: 10.1073/pnas.1911273116, PMID: 31818951 PMC6936689

[166] Sachs N de Ligt J Kopper O Gogola E Bounova G Weeber F . A living biobank of breast cancer organoids captures disease heterogeneity. Cell. (2018) 172:373–386.e10. doi: 10.1016/j.cell.2017.11.010, PMID: 29224780

[167] Ni K Luo T Culbert A Kaufmann M Jiang X Lin W . Nanoscale metal-organic framework co-delivers TLR-7 agonists and anti-CD47 antibodies to modulate macrophages and orchestrate cancer immunotherapy. J Am Chem Soc. (2020) 142:12579–84. doi: 10.1021/jacs.0c05039, PMID: 32658476

[168] Chen H Liu L Ma A Yin T Chen Z Liang R . Noninvasively immunogenic sonodynamic therapy with manganese protoporphyrin liposomes against triple-negative breast cancer. Biomaterials. (2021) 269:120639. doi: 10.1016/j.biomaterials.2020.120639, PMID: 33434714

[169] Du S Xiong H Xu C Lu Y Yao J . Attempts to strengthen and simplify the tumor vascular normalization strategy using tumor vessel normalization promoting nanomedicines. Biomater Sci. (2019) 7:1147–60. doi: 10.1039/C8BM01350K, PMID: 30648713

[170] Park SE Georgescu A Huh D . Organoids-on-a-chip. Science. (2019) 364:960–5. doi: 10.1126/science.aaw7894, PMID: 31171693 PMC7764943

[171] Poletti M Arnauts K Ferrante M Korcsmaros T . Organoid-based models to study the role of host-microbiota interactions in IBD. J Crohns Colitis. (2021) 15:1222–35. doi: 10.1093/ecco-jcc/jjaa257, PMID: 33341879 PMC8256633

[172] Heinrich MA Bansal R Lammers T Zhang YS Michel Schiffelers R Prakash J . 3D-bioprinted mini-brain: A glioblastoma model to study cellular interactions and therapeutics. Adv Mater. (2019) 31:e1806590. doi: 10.1002/adma.201806590, PMID: 30702785

[173] Kim E Choi S Kang B Kong J Kim Y Yoon WH . Creation of bladder assembloids mimicking tissue regeneration and cancer. Nature. (2020) 588:664–9. doi: 10.1038/s41586-020-3034-x, PMID: 33328632

[174] Grunewald L Lam T Andersch L Klaus A Schwiebert S Winkler A . A reproducible bioprinted 3D tumor model serves as a preselection tool for CAR T cell therapy optimization. Front Immunol. (2021) 12:689697. doi: 10.3389/fimmu.2021.689697, PMID: 34267756 PMC8276678

